# Placental growth factor promotes neural invasion and predicts disease prognosis in resectable pancreatic cancer

**DOI:** 10.1186/s13046-024-03066-z

**Published:** 2024-05-30

**Authors:** Andreas Göhrig, Georg Hilfenhaus, Friederike Rosseck, Martina Welzel, Benjamin Moser, Gianluca Barbone, Catarina Alisa Kunze, Johannes Rein, Gregor Wilken, Michael Böhmig, Thomas Malinka, Frank Tacke, Marcus Bahra, Katharina M. Detjen, Christian Fischer

**Affiliations:** 1 Department of Hepatology & Gastroenterology, Charité-Universitätsmedizin Berlin, Corporate Member of Freie Universität Berlin and Humboldt-Universität zu Berlin, Campus Virchow-Klinikum and Campus Charité Mitte, Berlin, Germany; 2 ECRC Experimental and Clinical Research Center, Charité-Universitätsmedizin Berlin, Corporate Member of Freie Universität Berlin and Humboldt-Universität zu Berlin, Berlin, Germany; 3 Department of Hematology, Oncology & Cancer Immunology, Charité-Universitätsmedizin Berlin, corporate member of Freie Universität Berlin and Humboldt-Universität zu Berlin, Campus Charité Mitte, Berlin, Germany; 4 Institute of Pathology, Charité-Universitätsmedizin Berlin, Corporate Member of Freie Universität Berlin and Humboldt-Universität zu Berlin, Campus Charité Mitte, Berlin, Germany; 5 Department of Pulmonology, Charité-Universitätsmedizin Berlin, Corporate Member of Freie Universität Berlin and Humboldt-Universität zu Berlin, Campus Charité Mitte, Berlin, Germany; 6 Present Address: Gastroenterologie an der Krummen Lanke, Fischerhüttenstraße 109, Berlin, 14163 Germany; 7 Department of Surgery, Charité-Universitätsmedizin Berlin, Corporate Member of Freie Universität Berlin and Humboldt-Universität zu Berlin, Campus Virchow-Klinikum, Berlin, Germany; 8 Department of Oncological Surgery and Robotics, Waldfriede Hospital, Berlin, Germany

**Keywords:** Pancreatic cancer, Placental growth factor (PlGF), Axon guidance molecule, Neural invasion, Nerves, Cancer neuroscience

## Abstract

**Background:**

Surgery represents the only curative treatment option for pancreatic ductal adenocarcinoma (PDAC), but recurrence in more than 85% of patients limits the success of curative-intent tumor resection. Neural invasion (NI), particularly the spread of tumor cells along nerves into extratumoral regions of the pancreas, constitutes a well-recognized risk factor for recurrence. Hence, monitoring and therapeutic targeting of NI offer the potential to stratify recurrence risk and improve recurrence-free survival. Based on the evolutionary conserved dual function of axon and vessel guidance molecules, we hypothesize that the proangiogenic vessel guidance factor placental growth factor (PlGF) fosters NI. To test this hypothesis, we correlated PlGF with NI in PDAC patient samples and functionally assessed its role for the interaction of tumor cells with nerves.

**Methods:**

Serum levels of PlGF and its soluble receptor sFlt1, and expression of PlGF mRNA transcripts in tumor tissues were determined by ELISA or qPCR in a retrospective discovery and a prospective validation cohort. Free circulating PlGF was calculated from the ratio PlGF/sFlt1. Incidence and extent of NI were quantified based on histomorphometric measurements and separately assessed for intratumoral and extratumoral nerves. PlGF function on reciprocal chemoattraction and directed neurite outgrowth was evaluated in co-cultures of PDAC cells with primary dorsal-root-ganglia neurons or Schwann cells using blocking anti-PlGF antibodies.

**Results:**

Elevated circulating levels of free PlGF correlated with NI and shorter overall survival in patients with PDAC qualifying for curative-intent surgery. Furthermore, high tissue PlGF mRNA transcript levels in patients undergoing curative-intent surgery correlated with a higher incidence and greater extent of NI spreading to tumor-distant extratumoral nerves. In turn, more abundant extratumoral NI predicted shorter disease-free and overall survival. Experimentally, PlGF facilitated directional and dynamic changes in neurite outgrowth of primary dorsal-root-ganglia neurons upon exposure to PDAC derived guidance and growth factors and supported mutual chemoattraction of tumor cells with neurons and Schwann cells.

**Conclusion:**

Our translational results highlight PlGF as an axon guidance factor, which fosters neurite outgrowth and attracts tumor cells towards nerves. Hence, PlGF represents a promising circulating biomarker of NI and potential therapeutic target to improve the clinical outcome for patients with resectable PDAC.

**Graphical Abstract:**

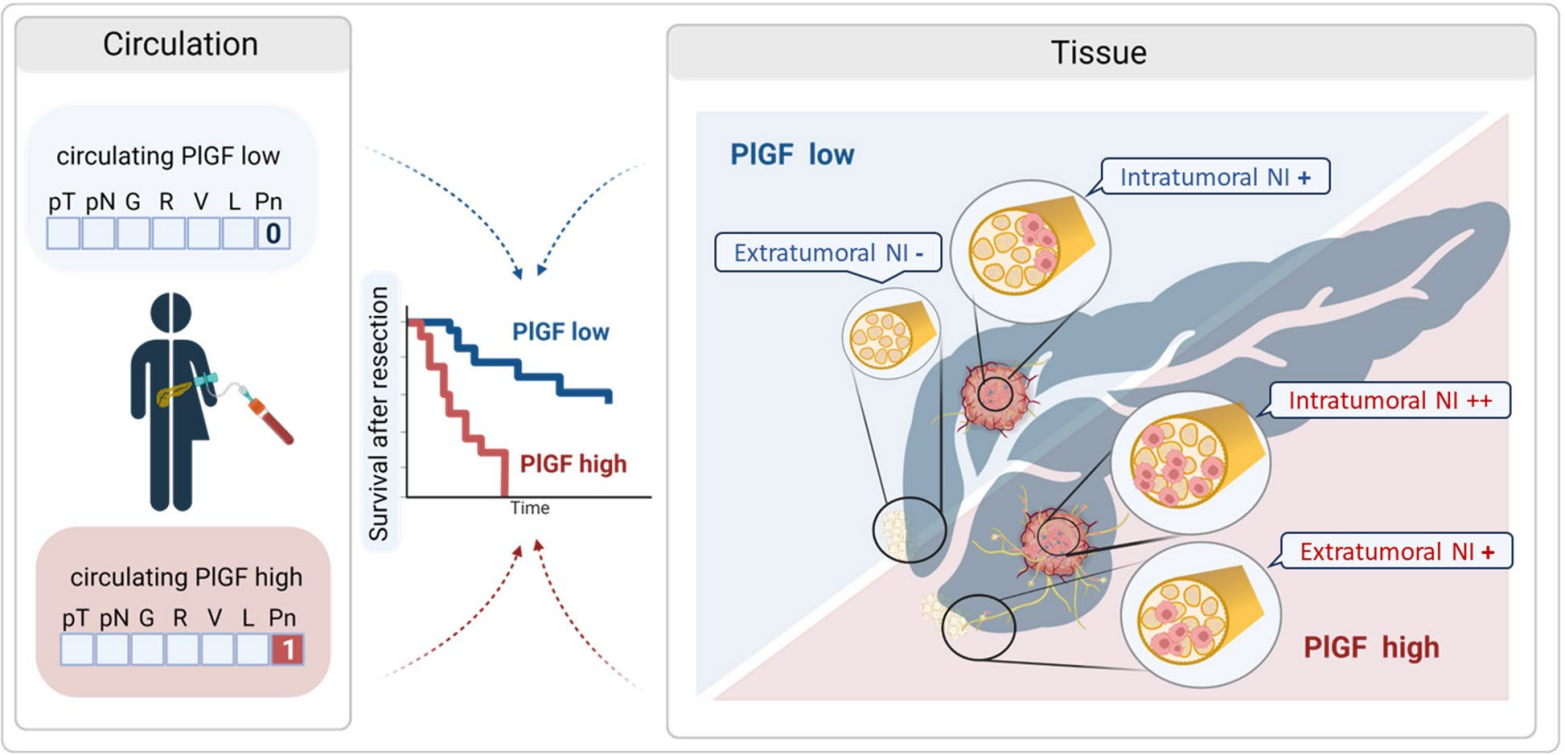

**Supplementary Information:**

The online version contains supplementary material available at 10.1186/s13046-024-03066-z.

## Background

Pancreatic ductal adenocarcinoma (PDAC) is projected to rank as second leading cause of cancer-related deaths in the Western world before 2030 [1]. At localized stages, surgery with (neo)adjuvant chemotherapy offers a curative treatment option, but eventually more than 85% of patients experience disease recurrence [2–4], with lymph node metastasis and neural invasion (NI) representing adverse prognostic factors [2, 5–7].

NI constitutes a key pathologic feature of PDAC and is perceived as a distinct route of tumor spread independent from lymphatic and hematogenous dissemination [5, 7]. NI is associated with an unfavorable clinical course, and with neuropathic pain, which frequently limits quality of life [5, 8, 9]. Current concepts of NI emphasize the reciprocal interaction between tumor cells and nerves, resulting in enhanced neural plasticity with axonal outgrowth towards tumor cells and tumor cell invasion along neurons and ensheathing Schwann cells [5, 7, 10]. Indeed, markers of neural plasticity such as increased nerve densities and neural hypertrophy are remarkably prevalent in PDAC [7, 11], with neurotrophins, cytokines, cell surface ligands and corresponding receptors being mechanistically implicated [5, 12–17].

Axon and vessel guidance factors uniquely qualify as candidate regulators of NI in PDAC [5, 12, 16, 18, 19]. Physiologically, axon guidance systems function as signaling cues to direct outgrowth and navigation of neurons in the developing brain [20]. As evolution progressed, they were co-opted for navigation of the emerging blood vessels [21], resulting in dual functions in neurogenesis and angiogenesis [22].

Placental growth factor (PlGF) is a member of the vascular endothelial growth factor (VEGF) family. PlGF and VEGF share many common structural features and use a similar repertoire of receptors. However, despite some synergistic functions, both growth factors cause distinct biological effects, which vary in a context-dependent manner and stem from their different mode of receptor binding and signaling properties [23]. Unlike VEGF, which binds VEGFR receptor-1 (VEGFR1) and VEGFR2, PlGF selectively binds VEGFR1, as well as Neuropilin-1 (Nrp1) and Nrp2 [23, 24]. Apart from enhancing VEGF-driven activity in angiogenesis, PlGF induces a distinct signaling pathway independent from VEGF [23]. PlGF is redundant for physiological vessel growth, but substantially contributes to pathological angiogenesis [23, 25, 26]. Accordingly, PlGF expression is hardly detectable under healthy conditions, but increased in malignancies, in which intratumoral and/or circulating PlGF levels correlated with vascularity, metastasis, recurrence and survival [23, 25, 27–29].

PlGF functions as a pleiotropic cytokine, which recruits and activates proangiogenic, desmoplastic and inflammatory cells within the tumor stroma [23, 28, 29]. Conversely, PlGF also transmits survival signals to tumor cells directly through Nrp1 [24].

The role of PlGF for nerve wiring is poorly understood, but similar to angiogenesis likely restricted to pathological conditions [23]. Indeed, induction of PlGF reduced ischemia induced cortical lesion size and functional deficits [30] and protected from retinal neural cell damage [31]. Moreover, induction of PlGF in Schwann cells upon peripheral nerve injury stimulated axonal regrowth, whereas genetic loss of PlGF accelerated Wallerian degeneration [32]. Furthermore, PlGF gene delivery in diabetic mice ameliorated sensory neuropathy by promoting regeneration of nerve fibers [33].

Here, we investigate the role of PlGF as a guidance cue which impacts the tumor cell-nerve crosstalk to promote NI and neural plasticity in PDAC.

## Material and methods

### Patients and PDAC biomaterials

Serum, tissue and clinicopathological information were obtained from individuals with PDAC treated at Charité-Universitätsmedizin Berlin from 1999–2022 (serum; detailed cohort description including age, gender and TNM classification are listed in Suppl. Table 1 and 2) or from 2010–2016 (tissue; Suppl. Table 3). Blood donors without history of malignant disease served as controls. Ethical approval was obtained from the institutional ethics committee (EA1/208/12, EA1/188/17; 62/2002; EA4/214/21; EA2/102/23).

Tumor staging with TNM classification using CT or MRI scans was performed at the time of blood sampling. In non-resectable locally advanced or metastatic disease, histopathological diagnosis was obtained by biopsies of primary tumors or metastases. In case of curative-intent surgery, histopathological features were obtained from pathology reports, including TNM, grading, neural invasion (Pn0, Pn1), lymph node metastasis (N0, N1, N2), angioinvasion (V0, V1), lymphangioinvasion (L0, L1) and histological tumor resection margin (R0, R1, R2). Tumor progression was based on multi-phasic computed tomography (CT) or magnetic resonance imaging (MRI) at follow-up staging visits at least every 3 months. Pain intensity was determined using a visual analogue scale (VAS 0–10), using the categories no pain (VAS 0), mild pain (VAS 1–2), moderate pain (VAS 3–6) and strong pain (VAS 7–10).

### Reagents

Generation and characterization of neutralizing antibodies to murine PlGF (5D11D4) and human PlGF (16D3) have been described [25, 27]. Recombinant human PlGF, NGF and GDNF, and Quantikine® ELISA kits for determination of human and mouse PlGF and human VEGF were from R&D Systems (Minneapolis, MN, USA). Circulating human PlGF and sFlt1 were determined using Elecsys® PlGF and sFlt1 immunoassays (Roche Diagnostics, Switzerland) as described [27].

### Cell lines

Cultures of human pancreatic carcinoma cell lines were used up to three months, before being replaced by frozen stocks generated shortly after receipt from the repositories, or after cell line authentication. ASPC1 (CRL-1682) cells were from ATCC (Manassas, USA); MiaPaCa (DSMZ no.: ACC 733), Panc1 (ACC 783), Capan-1 (ACC 244), Capan-2 (ACC 245), HUP-T3 (ACC 259) and DANG (ACC 249) cells were from DSMZ (Braunschweig, Germany) and maintained as described [34]. The immortal HPDE (human pancreatic duct epithelial) cell line H6c7 was obtained from the University Health Network (Ontario, Canada). Schwann cells and F11 neurons were a generous gift from Carmen Birchmeier and Fritz Rathjen (both Max-Delbrück-Center for Molecular Medicine, Berlin, Germany), respectively.

### Growth and migration assays

10^5^ cells were plated in 24-well-dishes, and cell numbers counted using a hemocytometer [34]. For migration assays, 2 × 10^5^ cells/insert were placed in serum-free medium in the upper chamber of 8 µm transwells (Corning®, Kaiserslautern, Germany) and allowed to migrate for 8-12 h towards 1% FCS added to the lower chamber. Tumor cell migration towards gradients from Schwann cells, cultured in the lower chamber for 16 h in DMEM (0.1% BSA), and vice versa, was determined. Migrated cells were stained with crystal-violet or DAPI and quantified by counting 5 standardized fields at 100 × magnification [12, 25, 27, 34]. Recombinant PlGF (50 ng/ml) and neutralizing anti-PlGF antibodies (100 µg/ml) were used as indicated.

### Histomorphometric analysis of neural invasion and plasticity

Parameters are based on evaluation of 30 mm^2^ areas within the tumor tissue (intratumoral) and adjacent to the tumor/organ margin (extratumoral) using Olympus BX46 microscope (Olympus, Tokyo, Japan), Moticam 3.0 camera and Motic Images Plus 2.0 ML software (Motic, Hong Kong, China). Neural plasticity was quantified by assessing number, caliber and area of intra- and extratumoral nerves allowing for a detailed representation of nerve density (number of nerves / cm^2^ tissue) and neural hypertrophy (total nerve area/ number of nerves). Neural invasion (NI) of intra- and extratumoral nerves was assessed by presence, localization (perineural vs. intraneural) and extent (perineural invasion score; NI dissemination score) of tumor cells within the neural space. Perineural invasion (PNI) score was assessed by determining the number of quadrants of the nerve circumference with PNI (score 0–4). NI dissemination score represents a semiquantitative assessment of “extensive” (score 2), “focal” (score 1) or “absent” (score 0) dissemination of neural invasion across standardized tissue sections. Nerve fraction was quantified by determining the percentage of tumor-invaded nerves per total numbers of intra- and extratumoral nerves examined. For an overview of the histomorphometric parameters determined refer to Fig. 4B and Supplement Fig. 4.

Morphometric analysis on serum-matched tissue samples in the prospective cohort encompassed the determination of the fraction of invaded nerves, the NI dissemination score and the determination of nerve density based on 20 high-power fields.

### Preparation of DRG primary cell cultures and enriched primary neurons

Dorsal root ganglia (DRG) were freshly isolated from newborn male C57Bl6 mice (Charles River Laboratories; Sulzfeld, Germany) as described [12]. Cultures of enriched primary neurons were isolated from DRGs using FudR (Fluorodeoxyuridine) treatment as described [12].

### Assays to study neural plasticity

Chemoattraction of neurons by PDAC derived neurotrophic factors was interrogated via transwell co-culture assays. Whole DRG primary cell cultures or enriched primary neurons were seeded in the upper chamber of FluoroBlok™ transwell inserts (Corning®, New York, USA). Filters with 1 μm pore size selectively allow outgrowing neurites but not neuron cell bodies to project through the transwell membranes towards chemoattractant gradients established by tumor cells or their conditioned media in the lower chamber. A light-tight polyethylene terephthalate membrane efficiently blocks light transmission from the FluoroBlok™ top chamber, allowing selective detection of CellTracker™ (Thermo Fisher, Darmstadt, Germany) labeled neurites at the bottom side of the membrane (using Leica DMI6000-B microscope). The number, mean length and density of neurites were measured. Alternatively, primary neurons were visualized by immunofluorescent β3-tubulin staining and subjected to morphometric analysis using NeuriteQuant® and AxioVision (Zeiss, Oberkochen, Germany) software [12]. Antibodies to PlGF were used as indicated.

To track the locomotion of individual neurons, primary neurons were co-cultured with tumor cells in separate patches divided by a 500 μm gap using IBIDI® inserts (Gräfeling, Germany). Neurons and tumor cells were selectively visualized by CellTracker™ [12].

### Tumor models

Local authorities approved animal experiments (protocol number: Reg 0101/10; LAGeSo Berlin, Germany). Female NMRI^nu/nu^ mice (6 weeks old) were from Charles River Laboratories (Sulzfeld, Germany). For orthotopic xenograft tumors, 10^6^ DANG cells (ACC 249) were injected into the head of the pancreas in 30 µl PBS using a 29-gauge needle following abdominal midline incision [12, 25, 34]. Starting 4 weeks after implantation, Gemcitabine (Gemzar®, Lilly, Germany) was i.p. administered twice a week at 125 mg/kg. After 7 weeks, mice were sacrificed, and primary tumors harvested for quantification of intratumoral levels of mouse and human PlGF by species-specific Quantikine® ELISA kits as described [12, 25].

### Statistics

Data are presented as mean ± SEM, circulating PlGF/sFlt1 as median with interquartile ranges. Significance was determined by Mann–Whitney test, Kruskal–Wallis test, Fisher's exact test and ANOVA using SPSS® (v18.0) and GraphPad® Prism (v10.0). Survival was estimated using the Kaplan–Meier method and Log-rank test. (*) *P* < 0.05 values were considered significant; all tests were two-sided.

For ELISA, quantitative RT-PCR, preparation of cell extracts and immunoblotting as well as immunohistochemistry refer to Supplemental Material and Methods.

## Results

### Elevated circulating PlGF/sFlt1 ratio is associated with neural invasion and predicts disease prognosis in PDAC

**Fig. 1:**
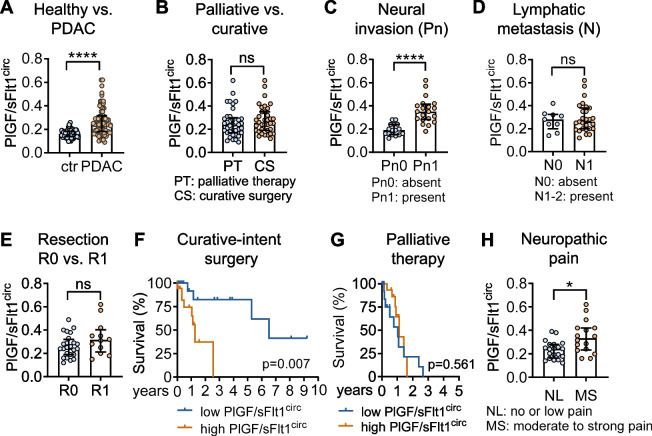
High circulating PlGF/sFlt1 serum ratio is associated with neural invasion and shorter survival in patients with PDAC undergoing curative-intent surgery. **A** PlGF/sFlt1^circ^ is elevated in PDAC patients (*n* = 73) compared to healthy controls (ctr; *n* = 79). **B** PlGF/sFlt1^circ^ does not differ between patients with non-resectable (locally advanced or metastatic tumors, PT; *n* = 37) and patients receiving curative-intent surgery (CS; *n* = 36). **C** In patients receiving curative-intent surgery, those with evidence of NI (Pn1; *n* = 20) exhibited higher PlGF/sFlt1^circ^ than patients without NI (Pn0; *n* = 16). **D** and **E** Comparable PlGF/sFlt1^circ^ ratios in patients with (**D** N1-2, *n* = 27) or without (N0; *n* = 9) lymph node metastasis and in patients with R0 (**E** *n* = 24) versus R1 resection (*n* = 12). **F** and **G** In Kaplan–Meier estimates PlGF/sFlt1^circ^ above cut-off correlates with shorter OS in patients with resectable PDAC (**F** HR: 4.05; 95% confidence interval: 1.22 to 13.48; Log-rank *p* = 0.007; *n* = 17 below and *n* = 19 above cut-off), but not with palliative disease (**G** HR: 0.845; 95% confidence interval: 0.33 to 2.19; *p* = 0.696; *n* = 20 below and *n* = 17 above cut-off). **H** Tumor-related pain was quantified using visual analogue scales (VAS 0–10) and grouped into no or low (0–3, *n* = 20), or moderate to strong pain (4–10, *n* = 16). Shown are PlGF/sFlt1^circ^ for the curative surgery cohort of patients. **A**-**E** and **H** scatter dot plots with median and interquartile range. *, *P* < 0.05; ****, *P* < 0.0001; ns, not significant

Given its release into the circulation, we retrospectively assessed circulating PlGF in patients with PDAC (please refer to Suppl. Table 1 for cohort description). Since soluble receptor Flt1 (sFlt1; sVEGFR1) binds PlGF [25], we also determined sFlt1 and calculated the ratio PlGF/sFlt1 (from here on referred to as PlGF/sFlt1^circ^) to estimate the fraction of unbound bioactive PlGF (please refer to Suppl. Figure 1A-H for individual results for PlGF and sFlt1). Compared to healthy controls, PlGF/sFlt1^circ^ was elevated in sera of PDAC patients (Fig. 1A), but did not differ between patients with advanced, non-resectable versus resectable tumors (Fig. 1B).

Notably, in patients undergoing curative-intent surgery, elevated PlGF/sFlt1^circ^ was associated with the occurrence of NI (Fig. 1C), but not with lymph node metastasis (Fig. 1D) nor histological tumor-free resection margins (Fig. 1E).

Based on ROC analyses, a cut-off at 0.251 PlGF/sFlt1^circ^ achieved optimal stratification of patients who were eligible for curative-intent surgery into subgroups with or without NI (Suppl. Figure 1I). Presurgical PlGF/sFlt1^circ^ above this cut-off was linked to shorter overall survival (OS; Fig. 1F) and stronger neuropathic pain (Fig. 1H). In the palliative situation PlGF/sFlt1^circ^ did not separate prognostic subgroups (Fig. 1G).

In contrast to PlGF/sFlt1^circ^, circulating VEGF in patients with PDAC did not differ from healthy controls, nor were VEGF levels associated with lymph node metastasis, NI, OS, and pain (Suppl. Figure 1 J-M). Moreover, PlGF/sFlt1^circ^ did not correlate with circulating VEGF levels in matched samples from the same patients (r_S_ = 0.261; *p* = ns).

### Circulating PlGF/sFlt1 ratio predicts neural invasion in a prospective cohort of PDAC

**Fig. 2:**
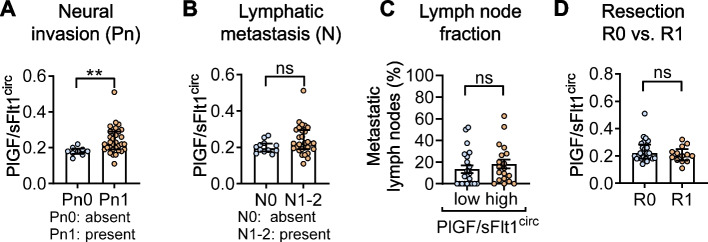
Circulating PlGF/sFlt1 ratio predicts neural invasion in a prospective cohort of PDAC patients undergoing curative-intent surgery. **A** PlGF/sFlt1^circ^ is elevated in patients with NI (*n* = 32) compared to patients without NI (*n* = 9). **B**-**D** PlGF/sFlt1^circ^ does not correlate with either incidence (**B**) or extent (**C**) of lymph node metastasis (**B** *n* = 12 for N0 and *n* = 29 for N1-2; **C** *n* = 22 for below and *n* = 19 for above median); or with R0 versus R1 resection margins (**D** *n* = 28 for R0, *n* = 13 for R1). **, *P* < 0.01; ns, not significant

The positive association between PlGF/sFlt1^circ^ and NI was corroborated in a prospective validation cohort of PDAC patients undergoing curative-intent surgery. As before, we found PlGF/sFlt1^circ^ elevated in patients with NI (Fig. 2A and Suppl. Figure 2), and tumors from patients with PlGF/sFlt1^circ^ above the cut-off at 0.251 all exhibited NI (*p* = 0.0195; Fisher’s exact test). Again, PlGF/sFlt1^circ^ did not correlate with the incidence and extent of lymph node metastasis (Fig. 2B and C) or tumor-free resection margins (Fig. 2D). Fittingly, a binominal logistic regression model identified PlGF/sFlt1^circ^ as predictive of NI in both, the retrospective and prospective cohort, whereas neither the status of lymphangioinvasion nor resection margins improved the model (Suppl. Table 4 and 5).

### PlGF and its receptor Nrp1 are expressed at the tumor-nerve interface in PDAC

**Fig. 3:**
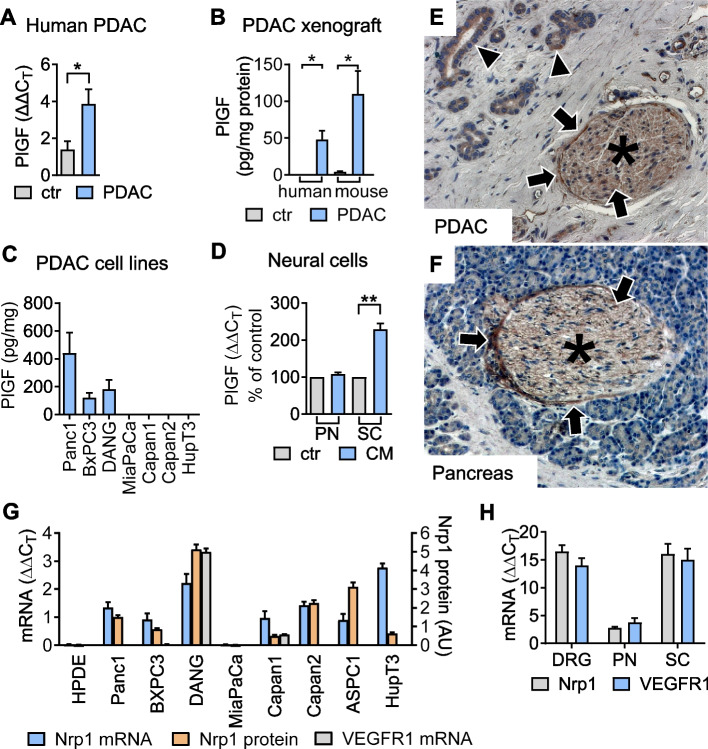
PlGF and its receptors are expressed at the tumor-nerve interface. **A** PlGF mRNA-transcript expression in human PDAC (*n* = 13) and healthy pancreas (ctr, *n* = 9). **B** Human (representing tumor cell derived) and murine (representing host derived) PlGF proteins in DANG orthotopic xenograft tumors (PDAC) and paired pancreas (ctr) determined using species-specific ELISA. **C** ELISA-based quantification of PlGF in supernatants of human PDAC cell line cultures (*n* = 3). **D** Primary neurons (PN) and Schwann cells (SC) from newborn mice were cultivated with control media (ctr) or conditioned media (CM) from MiaPaCa tumor cell cultures and PlGF mRNA expression determined (*n* = 3). Tumor cell conditioned supernatants induce PlGF expression in Schwann cells. **E** and **F** Representative IHC for the PlGF receptor Nrp1 in tissues of PDAC (**E**) and healthy pancreas (**F**). Intratumoral (in **E**) and intrapancreatic (in **F**) nerves are indicated by asterisks. Nrp1 expression in ductal epithelial cancer cells (arrowheads) and nerves (arrows). **G** and **H** mRNA transcripts and protein expression of Nrp1 and VEGFR1, respectively, in (**G**) human PDAC cell lines, the immortalized human pancreatic ductal epithelial cell line HPDE, as well as in (**H**) dorsal root ganglia (DRG), primary neurons (PN) and Schwann cells (SC; *n* = 3). *, *P* < 0.05; **, *P* < 0.01

Consistent with elevated PlGF/sFlt1^circ^ in PDAC patients, PlGF mRNA-transcript levels were increased in human PDAC tissues (Fig. 3A) and orthotopic xenograft tumors (Fig. 3B, and own previous data [25]) compared to the adjacent healthy pancreas. Both, tumor cells and tumor stroma constitute sources of PlGF, as determined by species-specific quantification of murine and human PlGF proteins in xenograft tumors (Fig. 3B) and human PDAC cell lines (Fig. 3C). High levels of PlGF mRNA transcripts correlated with a higher score of stromal inflammation and desmoplasia in human PDAC (Suppl. Figure 3A and B), consistent with expression of PlGF in tumor-associated macrophages and cancer-associated fibroblasts [23, 25, 28, 29]. Moreover, conditioned supernatants from PDAC cell cultures induced PlGF expression in primary Schwann cells, but not in primary neurons (Fig. 3D).

Consistent with previous reports on expression of Nrp1 in tumor and stromal cells in PDAC [29, 35, 36], Nrp1 immunoreactivity was found on tumor cells, parenchymal nerves and on vascular endothelial cells, but was only weakly present in epithelial cells of healthy pancreas (Fig. 3E and F). Correspondingly, human pancreatic ductal epithelial (HPDE) cells lack Nrp1 mRNA and protein expression, which contrasts with abundant Nrp1 mRNA transcripts and protein levels in most PDAC cell lines (Fig. 3G). Experimentally, we found interaction of PlGF with the receptor Nrp1 required and sufficient to stimulate clonal growth of PDAC cells (Suppl. Figure 3C and D).

Notably, Nrp1 mRNA transcripts were also present in murine primary neurons, Schwann cells, and dorsal root ganglia (DRG; Fig. 3H), as well as in F11 neuron cultures (not shown). In contrast, VEGFR1 mRNA transcripts were rarely detectable among human PDAC cell lines (Fig. 3G), but expressed in DRGs, primary neurons, F11 neurons and Schwann cells (Fig. 3H; and not shown), which is consistent with the reported stromal expression pattern in human PDAC tissues [37].

### Neural invasion of extratumoral nerves predicts early disease recurrence in PDAC

**Fig. 4:**
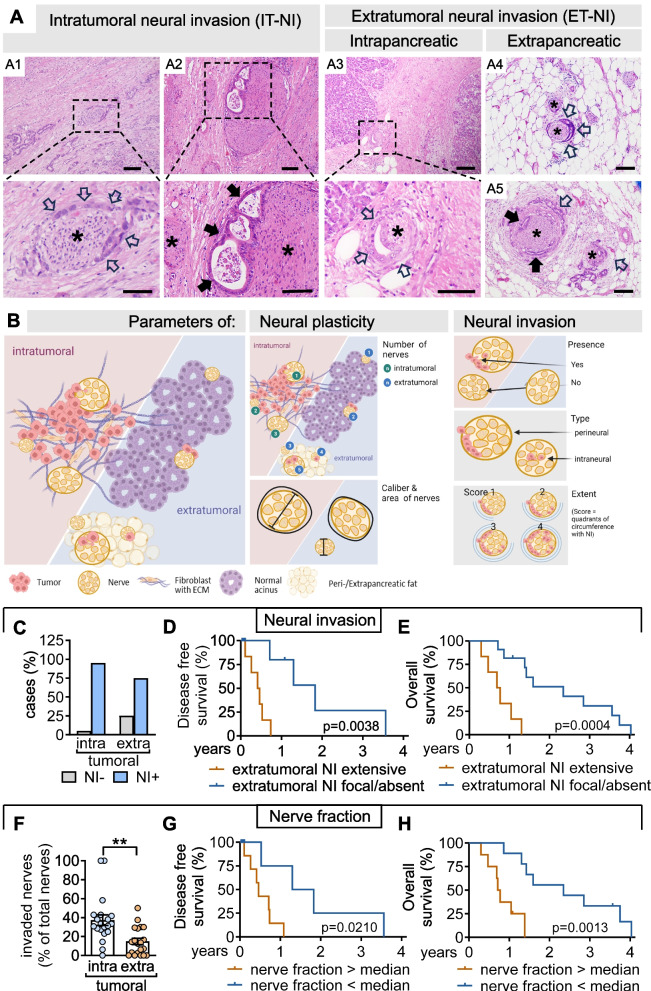
Morphologic parameters of neural invasion and plasticity: Neural invasion of extratumoral nerves predicts early disease recurrence and overall survival. **A** Representative IHC images and magnifications of intratumoral (A1 and A2) and extratumoral NI (A3-A5; scale bars, 200 μm). Shown are perineural invasion (PNI; open arrows) and intraneural invasion (INI; filled arrows) of intratumoral (in A1 and A2) and extratumoral (intrapancreatic in A3; extrapancreatic in A4 and A5) nerves (asterisks). **B** Illustration summarizing morphologic parameters of neural plasticity and invasion (created with BioRender.com). Neural plasticity is quantified by number, caliber and area of intra-/extratumoral nerves (middle panel). Neural invasion is assessed by presence, localization (perineural vs. intraneural) and extent (circumference; score 1–4) of tumor cells within the neural space (right panel). **C** Incidence of tumors with (NI +) or without (NI-) neural invasion (*n* = 20; *p* = 0.19, Fisher’s exact test) in PDAC specimens (intratumoral) and corresponding adjacent healthy pancreas (extratumoral). **D** and **E** Data on DSF and OS was available for *n* = 17 patients. A high neural dissemination score of extratumoral nerves is associated with shorter DFS (**D**) and OS (**E**) in patients receiving curative-intent surgery. Kaplan–Meier estimates depict DSF and OS of patients with extensive neural invasion of extratumoral nerves (score 2; *n* = 6) versus absent or focal neural invasion (score 0 and 1, respectively; *n* = 11; HR: 4.57; 95% confidence interval: 1.09 to 19.22; Log-rank *p* = 0.0038 in **D** HR: 4.63; 95% confidence interval: 1.00 to 21.52; Log-rank *p* = 0.0004 in **E**). **F** Percentage of tumor-invaded nerves per total nerves in PDAC specimens (intratumoral) and adjacent healthy pancreas (extratumoral). **G** and **H** High fraction of invaded extratumoral nerves is associated with shorter DFS (**G**) and OS (**H**) in patients receiving curative-intent surgery. Kaplan–Meier estimates depict DSF and OS of patients with extratumoral nerve fractions above (*n* = 8) or below (*n* = 9) median (HR: 3.29; 95% confidence interval: 0.95 to 11.39; Log-rank *p* = 0.0210 in **G;** HR: 3.93; 95% confidence interval: 1.06 to 14.58; Log-rank *p* = 0.0013 in **H**). **, *P* < 0.01

In routine pathology, NI is reported as a qualitative feature present in up to 90% of PDAC samples [2, 5, 9, 11]. To obtain a quantitative representation of NI, we performed morphometric analyses evaluating the presence, quality and area of tumor cell invasion of nerves, and the number and diameters of nerves as a measure of nerve density and hypertrophy (Fig. 4A and B and Suppl. Figure 4). Since NI can spread beyond the tumor boundaries, these parameters were recorded in standardized areas within the tumor (intratumoral; IT) and within the non-transformed pancreas outside the tumor margin (extratumoral; ET).

NI of intratumoral nerves (IT-NI) occurred in almost all patients, but involvement of extratumoral nerves affected only 2/3 of patients (ET-NI; Fig. 4C). The extent of NI widely varied between patients and between intra- versus extratumoral areas, as indicated by the fraction of tumor-invaded nerves per total nerves (Fig. 4F), which was lower in extratumoral when compared to intratumoral regions.

Clinically, the presence and extent of ET-NI translated into an unfavorable prognosis in patients undergoing curative-intent surgery. Indeed, strong involvement of extratumoral nerves, as reflected by an extensive ET-NI dissemination score (Fig. 4D and E, and Suppl. Figure 4) and high fractions of tumor-invaded extratumoral nerves (Fig. 4G and H) correlated with shorter disease-free survival (DFS) and OS.

### Expression of PlGF transcripts correlates with neural invasion of extratumoral nerves

**Fig. 5:**
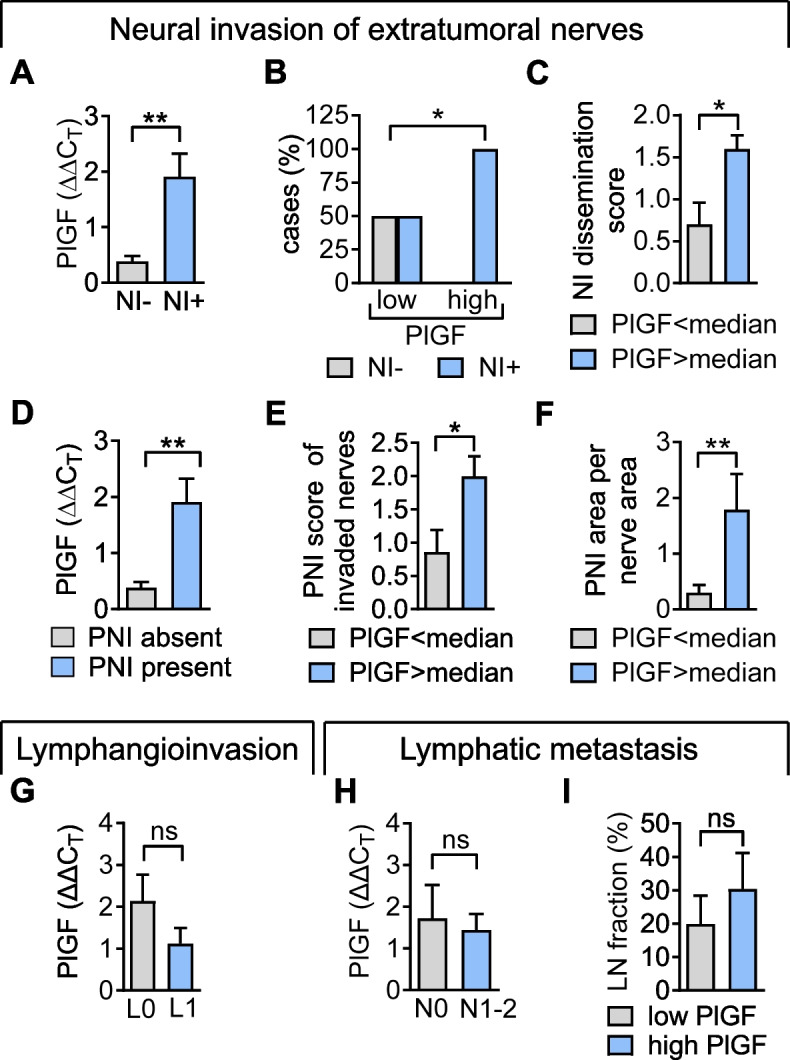
PlGF mRNA transcript levels correlate with the extent of neural invasion of extratumoral nerves. **A-I** Analyses refer to *n* = 20 PDAC samples that allowed for examination of extratumoral nerves. **A** PlGF mRNA transcript levels dependent on presence (NI +) or absence of neural invasion (NI-). **B** Incidence of NI in tumors with PlGF mRNA transcripts < median and > median. **C** Semiquantitative assessment of the NI dissemination as extensive (score 2), focal (score 1) or absent (score 0) in tumors with PlGF mRNA transcripts < median and > median. **D** PlGF mRNA transcript levels in tumors without (PNI absent) or with perineural invasion (PNI present). **E** Circumferential range of PNI was morphometrically determined and scored as 0 (absent), 1 (1/4 circumference), 2 (1/2 circumference), 3 (3/4 circumference), and 4 (whole circumference). Shown are PNI scores of affected nerves in tumors with PlGF mRNA transcripts < median and > median. **F** The PNI area fraction was determined by calculating the ratio of the PNI tumor cell area and the area of the corresponding nerve. **G** PlGF mRNA transcripts in tumors without (L0) and with (L1) lymphangioinvasion. **H** and **I,** Comparable PlGF mRNA expression in tumors with (N1-2) and without (N0) lymphatic metastasis (**H**) and, conversely, similar fractions of tumor infiltrated lymph nodes per total lymph nodes in tumors with PlGF mRNA transcripts < median and > median (**I**). *, *P* < 0.05; **, *P* < 0.01; ns, not significant

We then assessed whether PlGF mRNA transcript levels correlated with the incidence and extent of NI in human PDAC tissues. Given the obvious clinical impact (see Fig. 4), our analyses focused on PlGF in relation to NI of extratumoral nerves (Fig. 5A-F). Tumors with ET-NI exhibited higher PlGF transcript levels as compared to tumors in which ET-NI was absent (Fig. 5A). Strikingly, all tumors with PlGF transcript levels > median showed ET-NI and exhibited a higher NI dissemination score, whereas tumors with low PlGF transcript levels either completely lacked ET-NI (50% of cases; Fig. 5B) or exhibited only low NI dissemination scores (Fig. 5C). Moreover, correlation analysis demonstrated a positive correlation between PlGF transcript levels and the fraction of invaded nerves (r_S_ = 0.4996; *p* = 0.0294; Suppl. Figure 5F). Next, we related PlGF transcript levels to morphologic features of more advanced ET-NI, such as a wide circumferential range of perineural tumor cell invasion along the sheath of nerves (referred to as perineural invasion; PNI), and tumor cell invasion within the intraneural space of nerves, referred to as intraneural invasion (INI; illustrated in Fig. 4A and B). Tumors with ET-PNI exhibited higher mean PlGF transcript levels as compared to tumors without ET-PNI (Fig. 5D). Conversely, tumors with PlGF transcript levels > median exhibited a high ET-PNI score (Fig. 5E) and a larger ET-PNI area (Fig. 5F), as determined from the range of circumferential growth of tumor cells and the area of tumor cell clusters within the perineural sheath (scheme illustrated in Fig. 4B). Clinically, occurrence and extent of ET-INI both predicted early disease recurrence and a shorter OS (Suppl. Figure 5A-D). In sharp contrast, PlGF transcript levels did not correlate with either lymphangioinvasion (Fig. 5G), or incidence (Fig. 5H) or the extent of lymphatic metastasis (Fig. 5I).

### Circulating PlGF/sFlt1 ratio correlates with a more extended neural invasion

Given the correlation of tissue PlGF mRNA to several parameters that describe the extent of NI, we next asked, whether PlGF/sFlt1^circ^ also reflects the extent of NI in a quantitative way. Therefore, we quantified NI on the basis of the most pertinent morphometric parameters in serum-matched tissue samples in the prospective cohort. Subsequent analyses revealed a significant correlation between PlGF/sFlt1^circ^ and the global fraction of invaded nerves, when both intratumoral and extratumoral nerves were evaluated (r_S_ = 0.3244, *p* = 0.0411; Suppl. Figure 5E).

Taken together, our morphometric analyses link PlGF/sFlt1^circ^ and tissue PlGF expression to the presence and extent of NI in the retrospective and prospective PDAC cohorts and support the notion that PlGF/sFlt1^circ^ may serve as a quantitative serum biomarker of NI, prompting us to experimentally explore the function of PlGF at the tumor-nerve interface.

### PlGF mediates mutual chemoattraction between tumor cells and Schwann cells

**Fig. 6:**
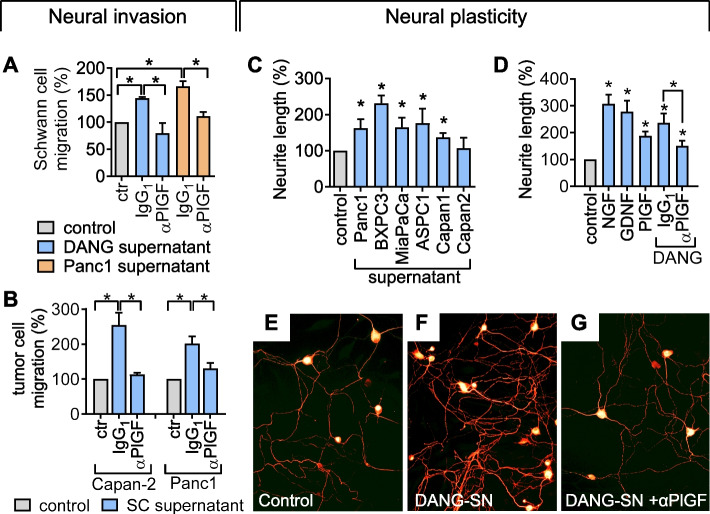
PlGF mediates mutual chemoattraction between tumor cells and Schwann cells and stimulates neurite outgrowth. **A** Neutralizing anti-PlGF antibodies inhibit directed migration of Schwann cells from the upper transwell chamber towards conditioned media from DANG or Panc1 monolayers (lower chamber) as compared to IgG_1_ control (*n* = 3). **B** Anti-PlGF inhibits directed migration of Panc1 and Capan-2 cells towards chemoattractant stimuli from conditioned Schwann cell supernatants placed in the lower chamber (*n* = 3). **C** and **D** Whole primary DRGs (containing neurons and Schwann cells) from newborn mice were incubated with supernatants from various PDAC cells (**C**) or medium containing recombinant nerve growth factor (NGF), glial-derived nerve growth factor (GDNF) and PlGF (**D**). Overall neurite length was determined using NeuroQuant® software based on selective staining of primary neurons for neuron-specific β3-tubulin (*n* = 3–5). PlGF stimulates neurite outgrowth, whereas neutralizing antibodies to PlGF secreted by DANG cells inhibit neurite outgrowth. **E**–**G** Representative images of β3-tubulin stained primary neurons cultured with control media (**E**), DANG supernatant (**F**) and DANG supernatant with anti-PlGF (**G**). *, *P* < 0.05

Schwann cells physiologically act as conduits for subsequent axonal outgrowth, but were shown to temporarily disengage from the perineural sheath and bridge the space towards tumor cell colonies in cancer, thus promoting NI [14, 38, 39]. We therefore determined the effects of PlGF on the mutual chemoattraction of Schwann cells and tumor cells. Conditioned media from DANG and Panc1 tumor cell lines, which endogenously secrete PlGF (Fig. 3C), enhanced the directed migration of Schwann cells, while inhibition of PlGF by using anti-PlGF, but not control IgG_1_ antibodies abrogated this effect (Fig. 6A). Vice versa, conditioned supernatant from Schwann cell cultures stimulated the directed migration of Panc1 and Capan-2 tumor cells (Fig. 6B), while blocking PlGF using anti-PlGF antibodies abolished the directed migration of PDAC cells towards chemoattractant released by Schwann cells (Fig. 6B). Thus, PlGF constitutes a bidirectional chemoattractant acting on tumor cells and Schwann cells.

### Tumor derived PlGF regulates neural plasticity

To assess neural plasticity, we performed co-culture assays combining PDAC cell lines with primary cell cultures from DRGs, freshly isolated primary neurons and Schwann cells, or with cultures of F11 hybridoma neurons.

First, primary neurons were co-cultured with either human pancreatic ductal epithelial (HPDE) or PDAC cells in separate patches using IBIDI® inserts (Suppl. Figure 6A-C), and nascent neurites were visualized at 48 h. Notably, neurite length increased upon co-culture with HupT3 PDAC cells as compared to HPDE cells. Moreover, stimulation of primary neuron cultures with conditioned media from HupT3 and DANG cell lines induced mRNA of the growth-associated-protein (GAP)-43, a marker of neural outgrowth and regeneration (Suppl. Figure 6D). Thus, ex vivo co-culture approaches capture aspects of neural plasticity in PDAC.

Second, whole DRG primary cell cultures instead of purified neurons were used, since the inclusion of Schwann cells more closely reflects the tumor-nerve interface in vivo. PDAC cell supernatants variably stimulated overall neurite length (Fig. 6C), as did recombinant PlGF and two established neurotrophic factors, nerve growth factor (NGF) or glial-derived neurotrophic factor (GDNF; Fig. 6D), used as positive controls.

Importantly, neutralizing antibodies to the endogenously produced PlGF in conditioned media from DANG cell cultures significantly reduced neurite length (Fig. 6D-G) and abrogated the directed migration of F11 neurons towards DANG cell supernatants (Suppl. Figure 6E). Thus, PlGF supported cancer-mediated neural plasticity.

### PlGF is induced following chemotherapy in vitro and in vivo

**Fig. 7:**
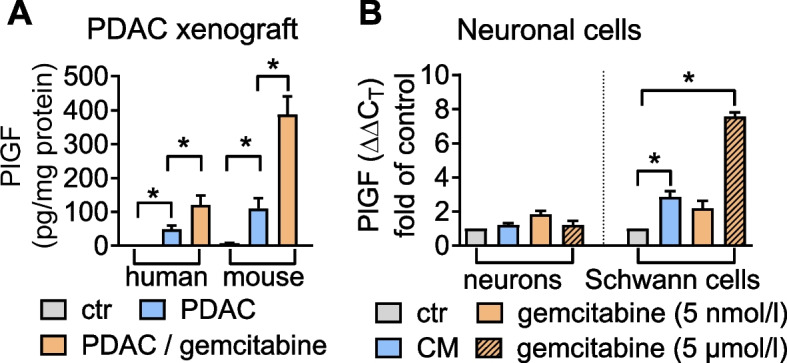
Chemotherapy induces PlGF expression within the neural compartment of PDAC. **A** Treatment of mice bearing orthotopic DANG tumors with the chemotherapeutic agent gemcitabine induces PlGF production by tumor epithelial cells (human) and stromal cells (mouse) as determined using species-specific ELISA. **B** Conditioned tumor cell supernatant (CM) and gemcitabine dose-dependently induce PlGF expression in Schwann cells (*n* = 3–5). *,*P* < 0.05

Clinical approaches to reduce tumor recurrence following curative-intent surgery focus on (neo)adjuvant chemotherapy. Therefore, we determined PlGF production in DANG xenograft tumors treated with the chemotherapeutic agent gemcitabine or vehicle. Chemotherapy increased PlGF expression in tumor epithelial cells and the stroma of PDAC xenografts (Fig. 7A). We also directly exposed primary neurons and Schwann cells to conditioned PDAC cell media supplemented with chemotherapy or vehicle and determined effects on PlGF. Chemotherapy concentration-dependently increased PlGF expression in Schwann cells, but not in primary neurons (Fig. 7B), confirming that chemotherapy enhances the availability of PlGF in the stromal compartment. In line with this observation, own previous data showed, that a combination treatment with anti-PlGF and chemotherapy potentiated growth inhibition of mouse orthotopic PDAC compared to monotherapies [25].

## Discussion

### The neurovascular link as conceptual basis for exploring NI in PDAC

Despite impressive improvements in surgical procedures and (neo)adjuvant treatments, PDAC recurrence remains almost inevitable [2, 3]. Hence, acting on our knowledge of NI as a major risk factor in PDAC postoperative tumor recurrence constitutes an unmet need, which led us to explore the role of the vessel and axon guidance factor PlGF in tumor-nerve interactions. The neurovascular link offers an appealing concept for NI based on analogies between guidance cues that function in angiogenesis and neurogenesis [5, 12, 16, 19–21]. Signals perceived hitherto as angiogenic may impact PDAC primarily by affecting the crosstalk to nerves. In analogy to their better studied role in angiogenesis, such signaling pathways may offer targetable vulnerabilities of NI.

### Functional role of PlGF in NI

**Fig. 8:**
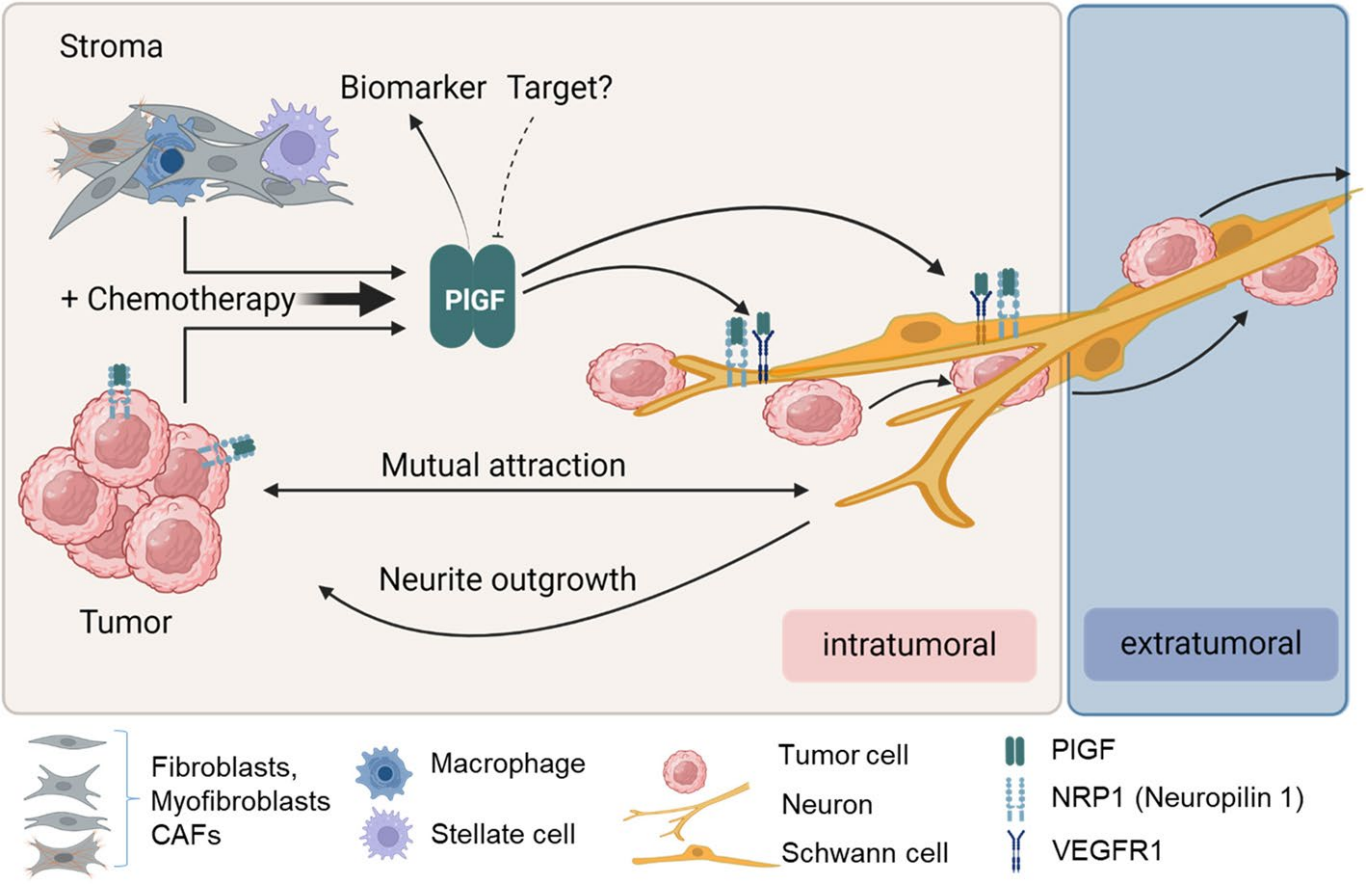
Cartoon summarizing the proposed model for the role of PlGF in neural invasion in PDAC. PlGF is expressed in various stromal cell types within the desmoplastic microenvironment and in tumor cells. Released PlGF then binds to and activates its corresponding receptors VEGFR1 and NRP1, which exhibit differential expression: NRP1 receptors are present on tumor cells, neurons and Schwann cells. Neurons and Schwann cells additionally express VEGFR1. PlGF-mediated activation of VEGFR1 and NRP1 stimulates the mutual attraction between tumor cells and neurons as well as Schwann cells, thereby supporting directed neurite outgrowth towards tumor cells and neural invasion. By directed migration tumor cells move along nerves from intratumoral into extratumoral regions of the adjacent normal pancreas thereby escaping curative-intent surgery. Chemotherapy induces PlGF production within the tumor microenvironment, thus creating a PlGF-rich tumor supportive niche which in turn may support neural invasion. Free circulating PlGF levels correlate with the presence of neural invasion and with the fraction of invaded nerves, providing a non-invasive biomarker of neural invasion. In addition, therapeutic targeting of PlGF is feasible and might be exploited to counteract neural invasion, and thereby reduce recurrence rates following curative-intent surgery. Created with BioRender

PlGF is best known as angiogenic growth factor, but evidently also serves a dual role as axon guidance cue [23, 31–33]. Our functional studies highlight two consequences of this axon guidance role: support of neural plasticity on one hand, and attraction of tumor cells towards nerves on the other hand (Fig. 8).

Specifically, we provide evidence that PlGF facilitates directional and dynamic changes in neurite outgrowth of primary neurons upon exposure to PDAC-derived guidance cues and supports mutual chemoattraction of tumor cells with neuronal cells and Schwann cells, respectively. The latter are receiving increasing attention as key players in NI of PDAC [14, 15, 38–40]. Our current results on neural plasticity align well with a previously reported role of VEGF and PlGF in cancer-related remodeling of nerves and enhancement of neuropathic pain: both growth factors induced nociceptive sensitization and augmented pain sensitivity through selective activation of VEGFR1, expressed in sensory neurons [41]. Strikingly, PlGF was even more potent than VEGF in inducing nociceptive hypersensitivity and neuropathic pain [41], which likely stems from differences in their distinct VEGFR1-binding and activating properties [23, 26]. Consistent with the proposed VEGFR1 dependent mechanisms, we found VEGFR1 expressed on neurons and Schwann cells, which responded to chemoattractant or trophic cues from PlGF. Moreover, we located tissue and cell-based expression of Nrp1 to both, the tumor cell and neuronal cell compartments in human PDAC, supporting the notion that PlGF signaling through Nrp1 might as well contribute to the neurotrophic effects of PlGF observed in our experimental ex vivo models of neural invasion and neurite outgrowth.

### Clinical assessment of NI and impact on (neo)adjuvant treatment situations

Findings from our experimental models translate well to our tissue-based observations in human PDAC. High PlGF mRNA-transcript levels correlate with higher incidence and greater extent of extratumoral NI, which predicted early disease recurrence and shorter survival in patients who underwent curative-intent surgery.

Despite the undisputed clinical impact of NI for tumor recurrence and OS in PDAC [5, 9], neither quantification of NI nor the assessment of extratumoral NI within the non-transformed tumor-distant pancreas has been implemented as standardized procedures in histopathological reports [42].

So far, only few studies provide comprehensive information on extratumoral NI [9]. Our current data add to the evidence that the severity of extratumoral NI is associated with reduced DFS and OS in PDAC patients receiving curative-intent surgery, thus underlining earlier proposals to implement quantitative assessment of NI in routine pathology reports [9, 43]. This is particularly important for more advanced tumors, in which high incidence of NI limits the prognostic value of a purely qualitative assessment of NI. Indeed, a recent study on a large PDAC cohort indicated a steep increase of NI between pT1 and pT2 classified tumors, such that larger PDACs (pT3 and pT4) present with NI in approximately 90% of cases, irrespective of prior neoadjuvant treatment [8].

Of the several aspects of NI that can be quantified, we envision that the evaluation of total nerves and invaded nerves in 20 high-power fields, each within the tumor boundaries and in the extratumoral area can be easily implemented in the routine evaluation of PDAC tissue by the pathologist. Indeed, the assessment of the nerve fraction as quantified by the ratio of invaded nerves per total nerves provided improved information on NI and disease-free and overall survival in our cohort, much in analogy to the determination of the lymph node fraction. With the advent of AI in digitized pathology [44], we expect a more comprehensive full morphometric determination of NI areas and neural hypertrophy will become available in the foreseeable future.

As nearly half of patients fail to complete or never receive standard of care adjuvant chemotherapy following surgery, current discussion favors upfront neoadjuvant chemotherapy for all patients with localized disease, based on several randomized clinical trials and meta-analyses with improved outcomes [45–48]. Yet, whether neoadjuvant chemotherapy has the capacity to target and reduce NI remains an unsolved issue, since incidence of NI is not reduced [8] and improved DFS likely results from delayed recurrence of distant metastasis whereas effects on local recurrence are minor [45]. Recent RNA-profiling analyses of resected PDAC samples with or without prior neoadjuvant chemoradiotherapy underscore this concern [49]: samples from patients receiving neoadjuvant treatment revealed more cells with activation of a neural-like progenitor gene program, possibly reflecting an enhanced capacity of tumor-nerve crosstalk. Of note, NRP1 was among the marker genes that specify the neural-like progenitor program [49], suggesting Nrp1 might be induced and enhance responsiveness to PlGF in tumors upon neoadjuvant treatment.

### Targeting PlGF to prevent PDAC recurrence

In our present study, chemotherapy induced expression of PlGF in Schwann cells and tumor epithelial cells, creating a putative scenario in which chemotherapy creates a PlGF-rich niche at the tumor-nerve interface, which in turn might frustrate efficient tumor cell eradication and facilitate disease recurrence. Two recently published reports already established that chemotherapy induced PlGF expression, which activated Nrp1-expressing cancer-associated fibroblasts, and thereby promoted desmoplasia and tissue stiffness in PDAC and cholangiocarcinoma [28, 29]. Consequently, reducing desmoplasia and stiffness via PlGF-blockade enhanced chemotherapy efficacy through vessel remodeling and improved vessel perfusion, and consequently hindered tumor growth [28, 29]. These studies did not comment on NI, and mouse models in general do not faithfully reflect neural invasion [17]. However, tumor stiffness per se can impinge on NI by inducing a pro-invasive and neurotrophic tumor phenotype [50]. As tumor stiffness supports NI, PlGF may also indirectly promote NI in PDAC via its effects on cancer-associated fibroblasts. Such indirect actions may complement the direct supportive effects of PlGF on the tumor cell-nerve interaction that we describe here. Together, these reports raise concerns that (neo)adjuvant treatments may create a microenvironment that favors NI. Consequently, therapeutic targeting of PlGF could provide a two-pronged approach to curtail NI and a beneficial adjunct to (neo)adjuvant treatments. Given the dual signaling of PlGF via Nrp1 and VEGFR1, inactivation of the ligand should be targeted, as has been studied for anti-PlGF antibodies and a PlGF/VEGF trap in preclinical mouse PDAC models [25, 29]. Moreover, the humanized monoclonal anti-PlGF antibody TB-403, which prevents PlGF binding to VEGFR1 and Nrp1, was shown effective in clinical trials [51, 52]. The low or undetectable expression of PlGF in healthy tissues suggests a favorable therapeutic window with lack of toxicities. Indeed, application of the anti-PlGF antibody TB-403 in heavily pretreated, relapsed pediatric medulloblastoma stabilized the disease in 7/11 children without significant toxicities [51].

### PlGF as biomarker for NI

Our retrospective and prospective cohorts of patients with PDAC undergoing curative-intent surgery delineated that PlGF/sFlt1^circ^ robustly reflected/predicted NI, recommending this easily accessible routine test as a circulating biomarker to differentiate patients with high or low probability of disease dissemination via NI. We also find that tissue PlGF mRNA-transcript levels and PlGF/sFlt1^circ^ both reflect quantitative parameters of NI determined by morphometry, although the exact correlations differed between our cohorts. While PlGF mRNA transcript levels correlated with the fraction of invaded extratumoral nerves, analogous analysis in the prospective cohort revealed a correlation of PlGF/sFlt1^circ^ with the global fraction of intra- and extratumoral nerves. One possible explanation can be found in the different composition of both cohorts. Indeed, in the prospective cohort, smaller pT1 and pT2 tumors comprised 75% of samples, whereas exclusively larger pT3 and pT4 tumors had been available for tissue based PlGF mRNA determination. Accordingly, the incidence of extratumoral NI in the prospective cohort was much lower and detected in only 30% of patients, thus precluding correlative analysis between PlGF/sFlt1^circ^ and the fraction of invaded extratumoral nerves. We acknowledge that a use of overlapping cohorts in the retrospective analyses on serum- and tissue samples would have been more informative, but we could only prospectively obtain matched tissue and serum samples.

Another limitation of our retrospective cohort stems from non-uniform therapies in patients who received either surgery only or surgery followed by adjuvant treatment. Consequently, we cannot conclude on the impact of adjuvant chemotherapy as potential confounder of our correlation between morphometric NI data and disease free and overall survival in our retrospective cohort.

### Future perspectives

Given the ongoing paradigm switch to upfront neoadjuvant chemotherapy in resectable PDAC, our results mandate a prospective evaluation of PlGF as a circulating and tissue-based biomarker for NI risk assessment. We envision this information can impinge on clinical decision making in several ways. For once, patients with a negligible risk of NI might be candidates for immediate surgery and subsequent adjuvant treatment rather than neoadjuvant treatment with a risk of providing an unfavorable, PlGF-rich tumor supportive niche in the perineural space. Second, patients with high risk of NI may benefit from targeting PlGF during adjuvant or neoadjuvant treatments. Third, high PlGF/sFlt1^circ^ levels may alert physicians to a high risk of early tumor recurrence, and accordingly aid challenging decisions about surgery in multimorbid patients with a high mortality risk.

Thus, further prospective and repeated measurements of PlGF/sFlt1^circ^ in well-defined clinical cohorts receiving (neo)adjuvant therapies are warranted in randomized trials to evaluate the value of baseline or treatment-induced PlGF/sFlt1^circ^ levels for risk allocation of patients to groups with high or low probability of NI. Such assessment of circulating PlGF/sFlt1^circ^ might particularly prove helpful in neoadjuvant situations, since pretreatment biopsies do not allow for tissue-based evaluation of NI and consequently access to NI as prognostic parameters would be lost. Furthermore, it is tempting to speculate that circulating PlGF/sFlt1^circ^ and tissue-based PlGF expression may reflect genetic subtypes of PDAC with enhanced capacities of tumor-nerve crosstalk allowing patients’ allocation to modified treatment algorithms.

Once validation has been obtained, PlGF directed therapies could be rapidly added to clinical trials in (neo)adjuvant treatments.

### Supplementary Information

Suppl. Tables.

Suppl. Figure 1.

Suppl. Figure 2.

Suppl. Figure 3.

Suppl. Figure 4.

Suppl. Figure 5.

Suppl. Figure 6.

Suppl. Material and Methods.

## Data Availability

Data confirming the results of this study are presented in the manuscript and are available from the corresponding author upon reasonable request.

## Abbreviations

DFS: Disease-free survival
DRG: Dorsal root ganglia
ET-NI: Extratumoral neural invasion
ET-PNI: Extratumoral perineural invasion
GDNF: Glial-derived neurotrophic factor
HPDE: Human pancreatic ductal epithelial cells
INI: Intraneural invasion
IT-NI: Intratumoral neural invasion
PDAC: Pancreatic ductal adenocarcinoma
PlGF: Placental growth factor
PNI: Perineural invasion
NGF: Nerve growth factor
NI: Neural invasion
Nrp1: Neuropilin-1
Nrp2: Neuropilin-2
OS: Overall survival
sFlt1: Soluble receptor Flt1
VAS: Visual analogue scale
VEGF: Vascular endothelial growth factor
VEGFR1: VEGF receptor-1

## References

[1] Rawla P, Sunkara T, Gaduputi V (2019). Epidemiology of pancreatic cancer: global trends, etiology and risk factors. World J Oncol.

[2] Collisson EA, Bailey P, Chang DK, Biankin AV (2019). Molecular subtypes of pancreatic cancer. Nat Rev Gastroenterol Hepatol.

[3] Mizrahi JD, Surana R, Valle JW, Shroff RT (2020). Pancreatic cancer. Lancet.

[4] Wood LD, Canto MI, Jaffee EM, Simeone DM (2022). Pancreatic cancer: pathogenesis, screening, diagnosis, and treatment. Gastroenterology.

[5] Bapat AA, Hostetter G, Von Hoff DD, Han H (2011). Perineural invasion and associated pain in pancreatic cancer. Nat Rev Cancer.

[6] Liang D, Shi S, Xu J, Zhang B, Qin Y, Ji S (2016). New insights into perineural invasion of pancreatic cancer: More than pain. Biochim Biophys Acta.

[7] Silverman DA, Martinez VK, Dougherty PM, Myers JN, Calin GA, Amit M (2021). Cancer-Associated Neurogenesis and Nerve-Cancer Cross-talk. Cancer Res.

[8] Crippa S, Pergolini I, Javed AA, Honselmann KC, Weiss MJ, Di Salvo F (2022). Implications of Perineural invasion on disease recurrence and survival after pancreatectomy for pancreatic head ductal Adenocarcinoma. Ann Surg.

[9] Schorn S, Demir IE, Haller B, Scheufele F, Reyes CM, Tieftrunk E (2017). The influence of neural invasion on survival and tumor recurrence in pancreatic ductal adenocarcinoma - A systematic review and meta-analysis. Surg Oncol.

[10] Xu W, Liu J, Zhang J, Lu J, Guo J (2023). Tumor microenvironment crosstalk between tumors and the nervous system in pancreatic cancer: Molecular mechanisms and clinical perspectives. Biochim Biophys Acta Rev Cancer.

[11] Ceyhan GO, Bergmann F, Kadihasanoglu M, Altintas B, Demir IE, Hinz U (2009). Pancreatic neuropathy and neuropathic pain–a comprehensive pathomorphological study of 546 cases. Gastroenterology..

[12] Göhrig A, Detjen KM, Hilfenhaus G, Korner JL, Welzel M, Arsenic R (2014). Axon guidance factor SLIT2 inhibits neural invasion and metastasis in pancreatic cancer. Cancer Res.

[13] Li X, Wang Z, Ma Q, Xu Q, Liu H, Duan W (2014). Sonic hedgehog paracrine signaling activates stromal cells to promote perineural invasion in pancreatic cancer. Clin Cancer Res.

[14] Demir IE, Boldis A, Pfitzinger PL, Teller S, Brunner E, Klose N (2014). Investigation of Schwann cells at neoplastic cell sites before the onset of cancer invasion. J Natl Cancer Inst.

[15] Deborde S, Wong RJ (2017). How Schwann cells facilitate cancer progression in nerves. Cell Mol Life Sci.

[16] Hung YH, Hou YC, Hsu SH, Wang LY, Tsai YL, Shan YS (2023). Pancreatic cancer cell-derived semaphorin 3A promotes neuron recruitment to accelerate tumor growth and dissemination. Am J Cancer Res.

[17] Wang X, Istvanffy R, Ye L, Teller S, Laschinger M, Diakopoulos KN (2023). Phenotype screens of murine pancreatic cancer identify a Tgf-alpha-Ccl2-paxillin axis driving human-like neural invasion. J Clin Invest..

[18] Jurcak NR, Rucki AA, Muth S, Thompson E, Sharma R, Ding D (2019). Axon Guidance Molecules Promote Perineural Invasion and Metastasis of Orthotopic Pancreatic Tumors in Mice. Gastroenterology..

[19] Fard D, Giraudo E, Tamagnone L (2023). Mind the (guidance) signals! Translational relevance of semaphorins, plexins, and neuropilins in pancreatic cancer. Trends Mol Med.

[20] Tessier-Lavigne M, Goodman CS (1996). The molecular biology of axon guidance. Science.

[21] Carmeliet P, Tessier-Lavigne M (2005). Common mechanisms of nerve and blood vessel wiring. Nature.

[22] Lange C, Storkebaum E, de Almodovar CR, Dewerchin M, Carmeliet P (2016). Vascular endothelial growth factor: a neurovascular target in neurological diseases. Nat Rev Neurol.

[23] Fischer C, Mazzone M, Jonckx B, Carmeliet P (2008). FLT1 and its ligands VEGFB and PlGF: drug targets for anti-angiogenic therapy?. Nat Rev Cancer.

[24] Snuderl M, Batista A, Kirkpatrick ND, de Almodovar CR, Riedemann L, Walsh EC (2013). Targeting placental growth factor/neuropilin 1 pathway inhibits growth and spread of medulloblastoma. Cell..

[25] Fischer C, Jonckx B, Mazzone M, Zacchigna S, Loges S, Pattarini L (2007). Anti-PlGF Inhibits Growth of VEGF(R)-Inhibitor-resistant tumors without affecting healthy vessels. Cell.

[26] Carmeliet P, Moons L, Luttun A, Vincenti V, Compernolle V, De Mol M (2001). Synergism between vascular endothelial growth factor and placental growth factor contributes to angiogenesis and plasma extravasation in pathological conditions. Nat Med.

[27] Hilfenhaus G, Göhrig A, Pape UF, Neumann T, Jann H, Zdunek D (2013). Placental growth factor supports neuroendocrine tumor growth and predicts disease prognosis in patients. Endocr Relat Cancer.

[28] Aoki S, Inoue K, Klein S, Halvorsen S, Chen J, Matsui A (2022). Placental growth factor promotes tumour desmoplasia and treatment resistance in intrahepatic cholangiocarcinoma. Gut.

[29] Kim DK, Jeong J, Lee DS, Hyeon DY, Park GW, Jeon S (2022). PD-L1-directed PlGF/VEGF blockade synergizes with chemotherapy by targeting CD141(+) cancer-associated fibroblasts in pancreatic cancer. Nat Commun.

[30] Liu H, Honmou O, Harada K, Nakamura K, Houkin K, Hamada H (2006). Neuroprotection by PlGF gene-modified human mesenchymal stem cells after cerebral ischaemia. Brain.

[31] Inoue Y, Shimazawa M, Nakamura S, Imamura T, Sugitani S, Tsuruma K (2014). Protective effects of placental growth factor on retinal neuronal cell damage. J Neurosci Res.

[32] Chaballe L, Close P, Sempels M, Delstanche S, Fanielle J, Moons L (2011). Involvement of placental growth factor in Wallerian degeneration. Glia.

[33] Murakami T, Imada Y, Kawamura M, Takahashi T, Fujita Y, Sato E (2011). Placental growth factor-2 gene transfer by electroporation restores diabetic sensory neuropathy in mice. Exp Neurol.

[34] Schulz P, Fischer C, Detjen KM, Rieke S, Hilfenhaus G, von Marschall Z (2011). Angiopoietin-2 drives lymphatic metastasis of pancreatic cancer. Faseb J.

[35] Ben Q, Zheng J, Fei J, An W, Li P, Li Z (2014). High neuropilin 1 expression was associated with angiogenesis and poor overall survival in resected pancreatic ductal adenocarcinoma. Pancreas.

[36] Matkar PN, Jong ED, Ariyagunarajah R, Prud'homme GJ, Singh KK, Leong-Poi H (2018). Jack of many trades: Multifaceted role of neuropilins in pancreatic cancer. Cancer Med.

[37] von Marschall Z, Cramer T, Hocker M, Burde R, Plath T, Schirner M (2000). De novo expression of vascular endothelial growth factor in human pancreatic cancer: evidence for an autocrine mitogenic loop. Gastroenterology.

[38] Deborde S, Gusain L, Powers A, Marcadis A, Yu Y, Chen CH (2022). Reprogrammed Schwann cells organize into dynamic tracks that promote pancreatic cancer invasion. Cancer Discov.

[39] Iacobuzio-Donahue CA (2023). The war on pancreatic cancer: progress and promise. Nat Rev Gastroenterol Hepatol.

[40] Pascual G, Dominguez D, Elosua-Bayes M, Beckedorff F, Laudanna C, Bigas C (2021). Dietary palmitic acid promotes a prometastatic memory via Schwann cells. Nature.

[41] Selvaraj D, Gangadharan V, Michalski CW, Kurejova M, Stosser S, Srivastava K (2015). A Functional Role for VEGFR1 expressed in peripheral sensory neurons in cancer pain. Cancer Cell.

[42] Zeng L, Guo Y, Liang J, Chen S, Peng P, Zhang Q (2014). Perineural invasion and TAMs in pancreatic ductal Adenocarcinomas: review of the original pathology reports using Immunohistochemical enhancement and relationships with clinicopathological features. J Cancer.

[43] Schmitd LB, Beesley LJ, Russo N, Bellile EL, Inglehart RC, Liu M (2018). Redefining perineural invasion: integration of biology with clinical outcome. Neoplasia.

[44] Borsekofsky S, Tsuriel S, Hagege RR, Hershkovitz D (2023). Perineural invasion detection in pancreatic ductal adenocarcinoma using artificial intelligence. Sci Rep.

[45] Groot VP, Blair AB, Gemenetzis G, Ding D, Burkhart RA, Yu J (2019). Recurrence after neoadjuvant therapy and resection of borderline resectable and locally advanced pancreatic cancer. Eur J Surg Oncol.

[46] Versteijne E, Suker M, Groothuis K, Akkermans-Vogelaar JM, Besselink MG, Bonsing BA (2020). Preoperative chemoradiotherapy versus immediate surgery for resectable and borderline resectable pancreatic cancer: results of the Dutch randomized phase III PREOPANC Trial. J Clin Oncol.

[47] van Dam JL, Janssen QP, Besselink MG, Homs MYV, van Santvoort HC, van Tienhoven G (2022). Neoadjuvant therapy or upfront surgery for resectable and borderline resectable pancreatic cancer: A meta-analysis of randomised controlled trials. Eur J Cancer.

[48] Ghaneh P, Palmer D, Cicconi S, Jackson R, Halloran CM, Rawcliffe C (2023). Immediate surgery compared with short-course neoadjuvant gemcitabine plus capecitabine, FOLFIRINOX, or chemoradiotherapy in patients with borderline resectable pancreatic cancer (ESPAC5): a four-arm, multicentre, randomised, phase 2 trial. Lancet Gastroenterol Hepatol.

[49] Hwang WL, Jagadeesh KA, Guo JA, Hoffman HI, Yadollahpour P, Reeves JW (2022). Single-nucleus and spatial transcriptome profiling of pancreatic cancer identifies multicellular dynamics associated with neoadjuvant treatment. Nat Genet.

[50] Han B, Guan X, Ma M, Liang B, Ren L, Liu Y, et al. Stiffened tumor microenvironment enhances perineural invasion in breast cancer via integrin signaling. Cell Oncol (Dordr). 2023. 10.1007/s13402-023-00901-x. Online ahead of print. 10.1007/s13402-023-00901-x PMC12974001 38015381

[51] Saulnier-Sholler G, Duda DG, Bergendahl G, Ebb D, Snuderl M, Laetsch TW (2022). A Phase I Trial of TB-403 in Relapsed Medulloblastoma, Neuroblastoma, Ewing Sarcoma, and Alveolar Rhabdomyosarcoma. Clin Cancer Res.

[52] Martinsson-Niskanen T, Riisbro R, Larsson L, Winstedt L, Stenberg Y, Pakola S (2011). Monoclonal antibody TB-403: a first-in-human, Phase I, double-blind, dose escalation study directed against placental growth factor in healthy male subjects. Clin Ther.

