# Supplementary material for: Placental growth factor promotes neural invasion and predicts disease prognosis in resectable pancreatic cancer
Source: J Exp Clin Cancer Res. 2024 May 30;43:153. doi: 10.1186/s13046-024-03066-z (PMC11138065; doi:10.1186/s13046-024-03066-z)
Supplement: Supplementary file 1 — Suppl. Tables. [file 13046_2024_3066_MOESM1_ESM.docx]

**Supplemental table 1:** Description of retrospective cohort for the determination of circulating PlGF.

| Characteristic | | Informative cases |  |  | Number of cases or value |
| --- | --- | --- | --- | --- | --- |
|  | *Age* | 73 | median |  | 66.9 (years) |
|  |  |  | range |  | 38.8 to 87.2 (years) |
|  | *Sex* | 73 | female |  | 39 |
|  |  |  | male |  | 34 |
|  | *Treatment* | 73 | palliative |  | 37 |
|  |  |  | curative intended surgery |  | 36 |
| *for curative surgery only* | *pTNM* (AJCC 8^th^) | 36 | pT | pT2 | 2 |
|  |  |  |  | pT3 | 34 |
|  |  |  | pN | N0 | 9 |
|  |  |  |  | N1-2 | 27 |
|  | *Angioinvasion* | 36 | V | V0 | 26 |
|  |  |  |  | V1 | 10 |
|  | *Lymphangioinvasion* | 36 | L | L0 | 17 |
|  |  |  |  | L1 | 19 |
|  | *Neural invasion* | 36 | Pn | Pn0 | 16 |
|  |  |  |  | Pn1 | 20 |
|  | *Resection margins* | 36 | R | R0 | 24 |
|  |  |  |  | R1 | 12 |
|  | *Grading* | 36 | G | G1 | 1 |
|  |  |  |  | G2 | 18 |
|  |  |  |  | G2-3 | 4 |
|  |  |  |  | G3 | 13 |

**Supplemental table 2:** Description of prospective cohort for the determination of circulating PlGF.

| Characteristic |  |  | Number of cases  or value | Percentage (%) |
| --- | --- | --- | --- | --- |
| *Age (years)* | median |  | 70.0 (years) |  |
|  | range |  | 41 to 86 (years) |  |
| *Sex* | female |  | 18 | 43.9 |
|  | male |  | 23 | 56.1 |
| *pTNM* | pT | pT1 | 6 | 14.6 |
|  |  | pT2 | 24 | 58.5 |
|  |  | pT3/4 | 10/1 | 24.4/2.4 |
|  | pN | N0 | 12 | 29.3 |
|  |  | N1 | 15 | 36.6 |
|  |  | N2 | 14 | 34.1 |
| *Grading* | G | G1 | 1 | 2.4 |
|  |  | G2 | 24 | 58.5 |
|  |  | G3 | 16 | 39.0 |
| *Angioinvasion* | V | V0 | 8 | 19.5 |
|  |  | V1 | 33 | 80.0 |
| *Lymphangioinvasion* | L | L0 | 24 | 58.5 |
|  |  | L1 | 17 | 41.5 |
| *Neural invasion* | Pn | Pn0 | 9 | 22.0 |
|  |  | Pn1 | 32 | 78.0 |
| *Resection margins* | R | R0 | 28 | 68.3 |
|  |  | R1 | 13 | 31.7 |

**Supplemental table 3:** Description of cohort for the morphometric analysis of neural invasion.

| Characteristic |  |  | Number of cases  or value | Percentage (%) |
| --- | --- | --- | --- | --- |
| *Age (years)* | median |  | 67 (years) |  |
|  | range |  | 50 to 85 (years) |  |
| *Sex* | female |  | 8 | 40.0 |
|  | male |  | 12 | 60.0 |
| *pTNM* | pT | pT1 | 0 | 0.0 |
|  |  | pT2 | 0 | 0.0 |
|  |  | pT3 | 17 | 85.0 |
|  |  | pT4 | 3 | 15.0 |
|  | pN | N0 | 7 | 35.0 |
|  |  | N1-2 | 13 | 65.0 |
| *Grading* | G | G1 | 0 | 0.0 |
|  |  | G2 | 8 | 40.0 |
|  |  | G3 | 12 | 60.0 |
| *Angioinvasion* | V | V0 | 17 | 85.0 |
|  |  | V1 | 3 | 15.0 |
| *Lymphangioinvasion* | L | L0 | 9 | 45.0 |
|  |  | L1 | 11 | 55.0 |
| *Resection margins* | R | R0 | 15 | 0.75 |
|  |  | R1 | 5 | 0.25 |

**Supplemental table 4:** Binominal logistic regression model for the prediction of neural invasion in the retrospective cohort of patients with PDAC undergoing curative-intent surgery.

|  | *Regression*  *coefficient B* | *SE* | *Wald* | *p* | *Odds ratio* | *95% CI for odds ratio* | |
| --- | --- | --- | --- | --- | --- | --- | --- |
|  |  |  |  |  |  | *Lower bound* | *Upper bound* |
| PlGF/sFlt1^circ^ (%) | 0.431 | 0.170 | 6.450 | 0.011 | 1.539 | 1.103 | 2.147 |
| Lymphangioinvasion (present vs. absent) | 4.405 | 1.916 | 5.285 | 0.022 | 81.837 | 1.914 | 3498.290 |
| Resection margins (R1/2 vs. R0) | 2.340 | 1.498 | 2.441 | 0.118 | 10.385 | 0.551 | 195.645 |
| Constant | -13.973 | 5.403 | 6.688 | 0.010 | 0.000 |  | |

**Supplemental table 5:** Binominal logistic regression model for the prediction of neural invasion in the prospective cohort of patients with PDAC undergoing curative-intent surgery.

|  | *Regression*  *coefficient B* | *SE* | *Wald* | *p* | *Odds ratio* | *95% CI for odds ratio* | |
| --- | --- | --- | --- | --- | --- | --- | --- |
|  |  |  |  |  |  | *Lower bound* | *Upper bound* |
| PlGF/sFlt1^circ^ (%) | 0.361 | 0.162 | 4.934 | 0.026 | 1.434 | 1.052 | 1.953 |
| pT status (1, 2, 3&4 pooled) | 0.037 | 0.715 | 0.003 | 0.958 | 1.038 | 0.256 | 4.215 |
| Lymphangioinvasion (present vs. absent) | 2.037 | 1.787 | 1.299 | 0.254 | 7.666 | 0.231 | 254.663 |
| Resection margins (R1/2 vs. R0) | 1.149 | 1.790 | 0.412 | 0.521 | 3.154 | 0.094 | 105.418 |
| Constant | -6.867 | 3.506 | 3.836 | 0.050 | 0.001 |  | |
