# Supplementary material for: Placental growth factor promotes neural invasion and predicts disease prognosis in resectable pancreatic cancer
Source: J Exp Clin Cancer Res. 2024 May 30;43:153. doi: 10.1186/s13046-024-03066-z (PMC11138065; doi:10.1186/s13046-024-03066-z)
Supplement: Supplementary file 2 — Suppl. Figure 1. [file 13046_2024_3066_MOESM2_ESM.pdf]

Suppl. Fig. 1

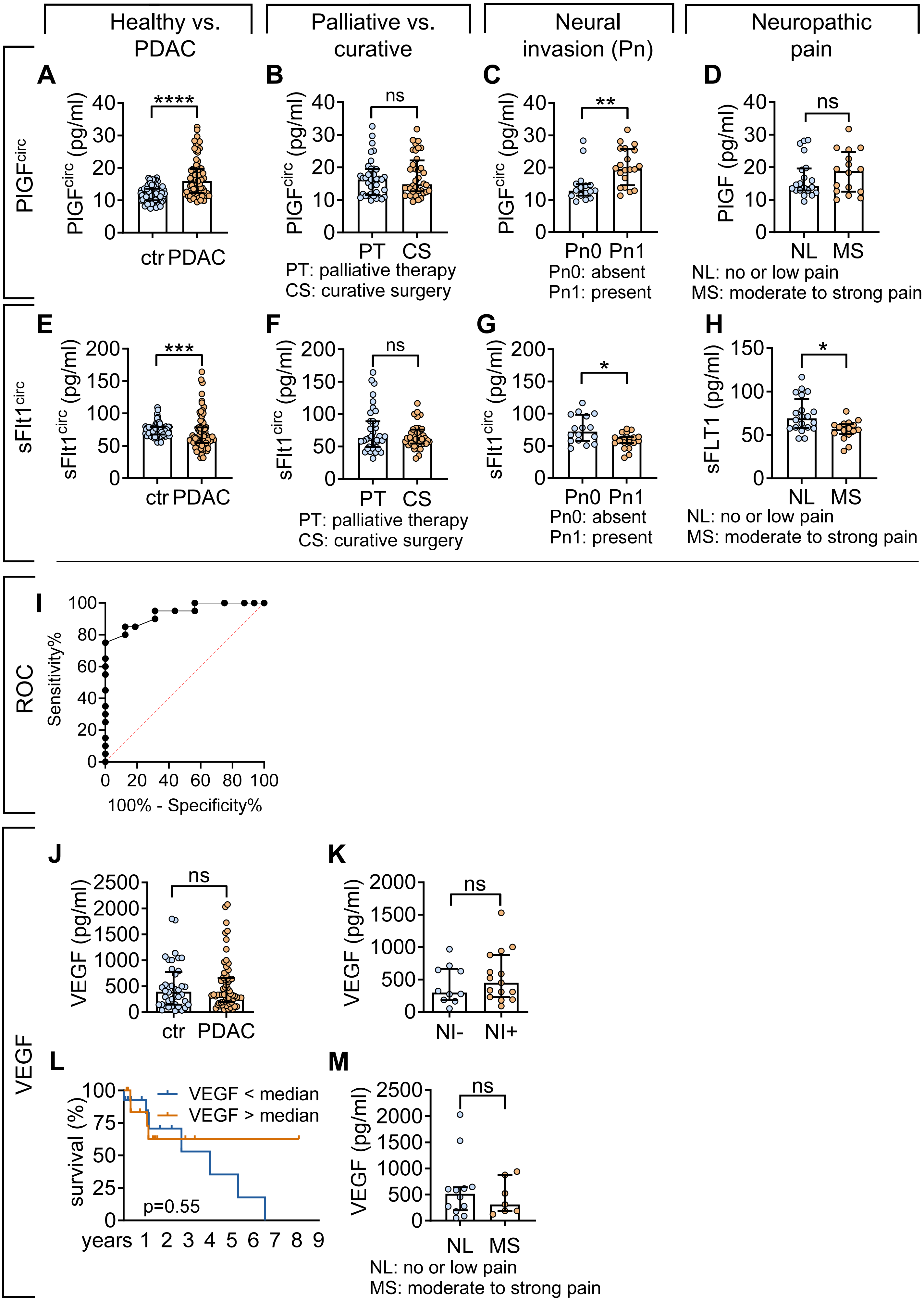

Healthy vs. PDAC
Palliative vs. curative
Neural invasion (Pn)
Neuropathic pain
PlGFcirc
A
PlGFcirc (pg/ml)
****
ctr PDAC
B
ns
PT CS
PT: palliative therapy
CS: curative surgery
C
**
Pn0 Pn1
Pn0: absent
Pn1: present
D
PlGF (pg/ml)
ns
NL MS
NL: no or low pain
MS: moderate to strong pain
sFlt1circ
E
sFlt1circ (pg/ml)
***
ctr PDAC
F
ns
PT CS
PT: palliative therapy
CS: curative surgery
G
*
Pn0 Pn1
Pn0: absent
Pn1: present
H
sFLT1 (pg/ml)
*
NL MS
NL: no or low pain
MS: moderate to strong pain
ROC
I
Sensitivity%
100% - Specificity%
VEGF
J
VEGF (pg/ml)
ns
ctr PDAC
K
ns
NI- NI+
L
survival (%)
VEGF < median
VEGF > median
p=0.55
years
M
ns
NL MS
NL: no or low pain
MS: moderate to strong pain

Suppl. Figure 1: *Evaluation of circulating PlGF and sFlt1 serum levels in PDAC patients. In contrast, VEGF is not associated with neural invasion and neuropathic pain and does not predict disease prognosis in patients with PDAC following curative-intent surgery.*

A, PlGF^{circ} is elevated in PDAC patients (n=73) compared to healthy controls (ctr; n=79). **B,** PlGF^{circ} does not differ among subgroups of patients with non-resectable (locally advanced or metastatic tumors; PT, n=37) and patients receiving curative-intent surgery (CS; n=36). **C,** In the CS cohort, median PlGF^{circ} is elevated in patients with NI (Pn1; n=20) compared to patients without NI (Pn0; n=16). **A-C** show scatter dot plots with median and interquartile range. **D,** Tumor-related pain was quantified using visual analogue scales (VAS 0-10) and grouped into no (0), low (1-3), moderate (4-6), and strong pain (7-10). Shown are PlGF^{circ} in the curative surgery cohort (CS) of patients reporting no or low (n=20) versus moderate to strong pain (n=16). **E,** sFlt1^{circ} is reduced in PDAC patients (n=73) compared to healthy controls (ctr; n=79). **F,** sFlt1^{circ} does not differ among subgroups of patients with non-resectable (locally advanced or metastatic tumors; PT, n=37) and patients receiving curative-intent surgery (CS; n=36). **G,** In the CS cohort, median sFlt1^{circ} is reduced in patients with NI (Pn1; n=20) compared to patients without NI (Pn0; n=16). **H,** Tumor-related pain was quantified (accordingly as referred to in **D**). Shown are sFlt1^{circ} in patients (CS cohort) reporting no or low (n=20) versus moderate to strong pain (n=16). **I,** Panel depicts ROC curve of PlGF/sFlt1^{circ} (AUC=0.934; 95% confidence interval: 0.858-1.000). Analysis displayed 85.0% sensitivity and 87.5% specificity for the discrimination between patients without and patients with NI at a cut-off PlGF/sFlt1^{circ} serum ratio of 0.251. **J,** Elevated VEGF serum levels in the overall cohort of PDAC patients (n=61) as compared to healthy controls (ctr; n=40). Shown is the scatter dot plot with the median and interquartile range; Mann-Whitney test. **K,** VEGF serum levels in patients with NI (NI+; n=15) and in patients without NI (NI-; n=10; Mann-Whitney test). **L,** Shown are Kaplan-Meier estimates of overall survival in patients with serum levels of VEGF <median (n=47) or VEGF >median (n=47), respectively, undergoing curative-intent surgery for resectable PDAC (HR: 1,44; 95% confidence interval: 0.432 to 4.7, Log-rank p=0.55). **M,** Pain was quantified using visual analogue scales (VAS 0-10) and grouped into no (0), low (VAS 1-3), moderate (VAS 4-6), and strong pain (VAS 7-10). Shown are circulating VEGF levels in the curative surgery cohort (CS) of patients reporting no or low (n=12) versus moderate to strong pain (n=7). *, P<0.05; **, P<0.01; ***, P<0.001; ****, P<0.0001; ns, not significant.
