# Supplementary material for: Placental growth factor promotes neural invasion and predicts disease prognosis in resectable pancreatic cancer
Source: J Exp Clin Cancer Res. 2024 May 30;43:153. doi: 10.1186/s13046-024-03066-z (PMC11138065; doi:10.1186/s13046-024-03066-z)
Supplement: Supplementary file 3 — Suppl. Figure 2. [file 13046_2024_3066_MOESM3_ESM.pdf]

Suppl. Fig. 2

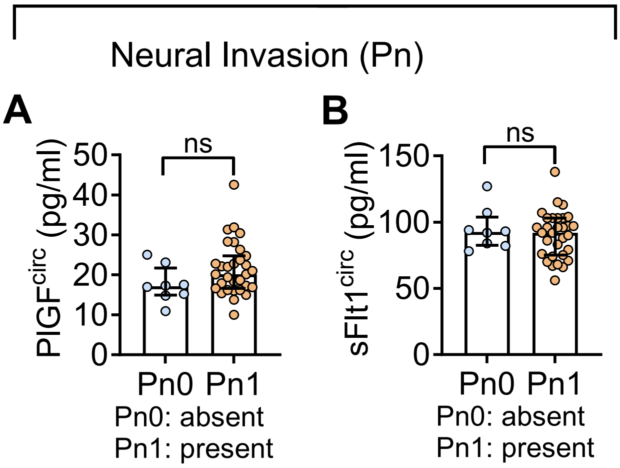

Suppl. Figure 2: *Prospective evaluation of PlGF ^{circ} and sFlt1^{circ} in a cohort of PDAC patients undergoing curative-intent surgery.*

A and **B,** Evaluation of PlGF^{circ} and sFlt1^{circ} in a prospective cohort of PDAC patients undergoing curative-intent surgery. Shown is median PlGF^{circ} (**A**) and median sFlt1^{circ} (**B**) of patients with NI (n=31) compared to patients without NI (n=8). ns, not significant.
