# Supplementary material for: Placental growth factor promotes neural invasion and predicts disease prognosis in resectable pancreatic cancer
Source: J Exp Clin Cancer Res. 2024 May 30;43:153. doi: 10.1186/s13046-024-03066-z (PMC11138065; doi:10.1186/s13046-024-03066-z)
Supplement: Supplementary file 4 — Suppl. Figure 3. [file 13046_2024_3066_MOESM4_ESM.pdf]

Suppl. Fig. 3

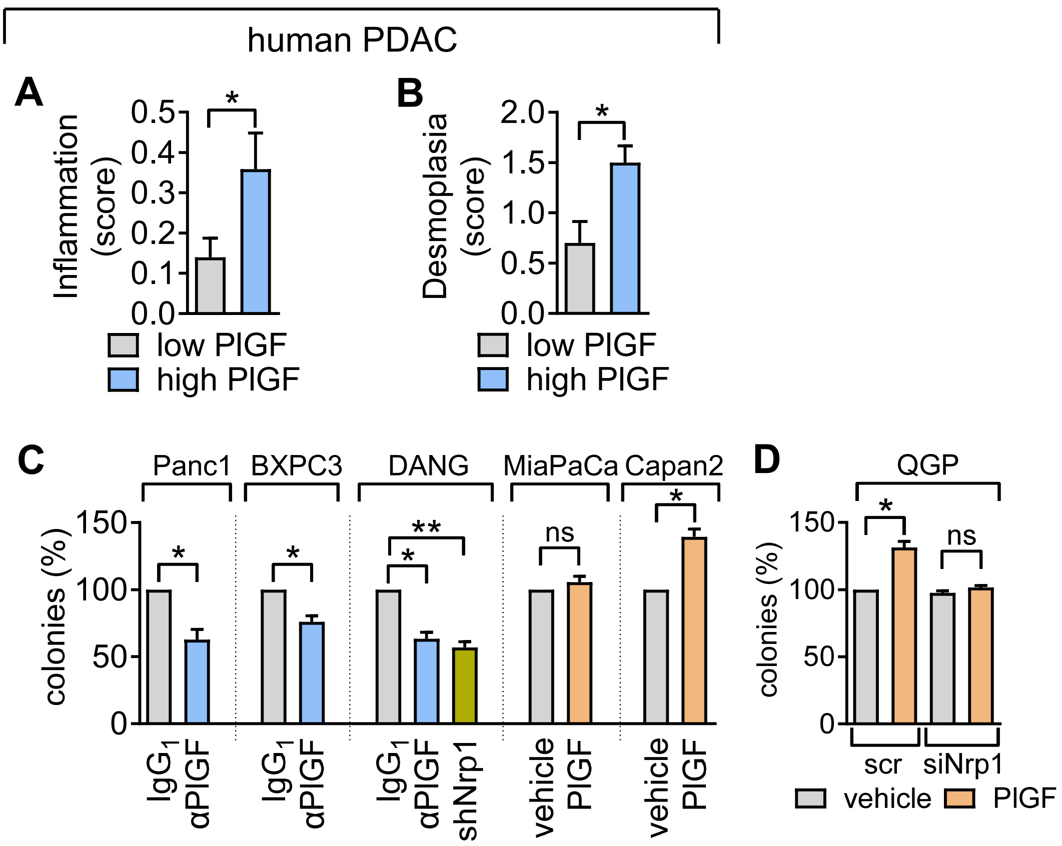

***Suppl. Figure 3:** PlGF correlates with desmoplasia and inflammation within the tumor stroma and stimulates colony formation of PDAC tumor cells via Nrp1.*

A and **B,** Semiquantitative morphometric analysis of inflammation and desmoplasia in human PDAC specimens. Shown are inflammation (**A**) and desmoplasia scores (**B**) as mean±SEM in tumors allocated to groups with PlGF mRNA-transcript levels <median or >median (n=20). Inflammation score defined as absence (0) or presence of perineural inflammatory cells (1). The extent of desmoplasia was scored based on the area covered by desmoplastic stroma using the semiquantitative score of 0 (absent), 1 (low) and 2 (high). **C,** Human PDAC cell lines were subjected to HTCA assays and the effects of anti-PlGF antibodies, Nrp1-receptor knock-down (using shRNA) and PlGF (100 ng/ml) on clonal growth determined. Disruption of autocrine PlGF stimulation using neutralizing anti-PlGF antibodies reduced clonal growth in VEGFR1-deficient but Nrp1-competent Panc1 and BXPC3 cells. In DANG cells neutralizing endogenous PlGF reduced clonal growth equal to knockdown of the Nrp1 receptor. PlGF stimulates colony formation of Nrp1-expressing Capan-2 (**C**) and QGP (**D**) cells but not of MiaPaCa cells (**C**), which lack Nrp1. **D,** Loss of responsiveness to PlGF in the VEGFR1-deficient pancreatic neuroendocrine tumor cell line QGP with siRNA-mediated knockdown of Nrp1 as compared to scrambled controls (scr; n=3-5). *, P<0.05; **, P<0.01; ns, not significant.
