# Supplementary material for: Placental growth factor promotes neural invasion and predicts disease prognosis in resectable pancreatic cancer
Source: J Exp Clin Cancer Res. 2024 May 30;43:153. doi: 10.1186/s13046-024-03066-z (PMC11138065; doi:10.1186/s13046-024-03066-z)
Supplement: Supplementary file 5 — Suppl. Figure 4. [file 13046_2024_3066_MOESM5_ESM.pdf]

Suppl. Fig. 4

Parameter	Calculation	Relates to
Nerve density	Number of nerves / cm^2 tissue	Plasticity
Neural hypertrophy	Total nerve area / number of nerves	Plasticity
Fraction of intratumoral invaded nerves	Number of tumor cell invaded intratumoral nerves / total number of intratumoral nerves	Invasion
Fraction of extratumoral invaded nerves	Number of tumor cell invaded extratumoral nerves / total number of extratumoral nerves	Invasion
PNI score	Number of quadrants of nerve circumference with PNI (score 0-4; illustrated in Fig. 4B)	Invasion
NI dissemination score	Dissemination of NI across tissue section; semiquantitative assessment of "extensive" (score 2), "focal" (score 1) or "absent" (score 0)	Invasion

Suppl. Figure 4: *Overview of calculated parameters applied to characterize and quantify extend of neural plasticity and neural invasion throughout the study.*
