# Supplementary material for: Placental growth factor promotes neural invasion and predicts disease prognosis in resectable pancreatic cancer
Source: J Exp Clin Cancer Res. 2024 May 30;43:153. doi: 10.1186/s13046-024-03066-z (PMC11138065; doi:10.1186/s13046-024-03066-z)
Supplement: Supplementary file 6 — Suppl. Figure 5. [file 13046_2024_3066_MOESM6_ESM.pdf]

Suppl. Fig. 5

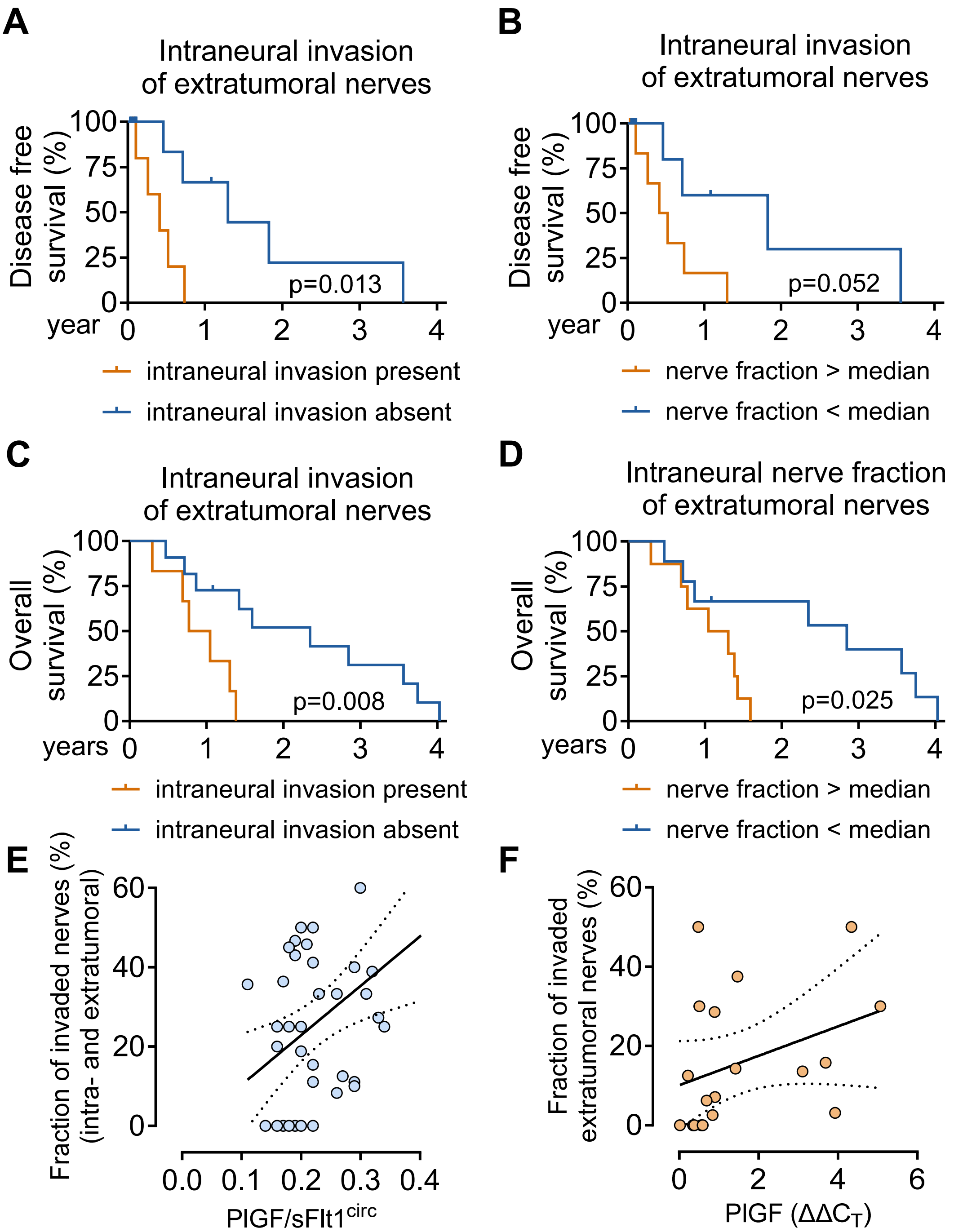

Suppl. Figure 5: *Intraneural invasion of extratumoral nerves predicts early disease recurrence and shorter overall survival (**A**-**D**), and PlGF correlates to the extent of neural invasion **(E-F)**.*

A and **B,** Intraneural invasion was quantified in extratumoral nerves. Kaplan-Meier depicts disease free survival in the subgroups of patients with (n=6) or without (n=11) intraneural nerve invasion of extratumoral nerves (HR: 3.87; 95% confidence interval: 0.84 to 17.97; Log-rank p=0.0126 in **A**), and in patients allocated to groups with intraneural nerve fraction of extratumoral nerves <median (n=9) and nerve fraction >median (n=8; HR: 3.03 for afflicted nerve fraction >median; 95% confidence interval: 0.81 to 11.29; Log-rank p=0.0521 in **B**). **C** and **D,** Analogous analyses for overall survival (HR: 3.30; 95% confidence interval: 0.85 to 12.83; Log-rank p=0.0075 in **C**; and HR: 2.63 for afflicted nerve fraction >median; 95% confidence interval: 0.88 to 7.88; Log-rank p=0.0246 in **D**). **E,** Positive correlation between PlGF/sFlt^{circ} and the global fraction of invaded nerves in 20 high-power fields (HPF) in the prospective cohort (Spearman r=0.324, p=0.041, n=40=. **F,** Positive correlation of PlGF mRNA ($\Delta\Delta C_T$) with the fraction of invaded extratumoral nerves (Spearman r=0.49, p=0.029, n=19).
