# Supplementary material for: Placental growth factor promotes neural invasion and predicts disease prognosis in resectable pancreatic cancer
Source: J Exp Clin Cancer Res. 2024 May 30;43:153. doi: 10.1186/s13046-024-03066-z (PMC11138065; doi:10.1186/s13046-024-03066-z)
Supplement: Supplementary file 7 — Suppl. Figure 6. [file 13046_2024_3066_MOESM7_ESM.pdf]

Suppl. Fig. 6

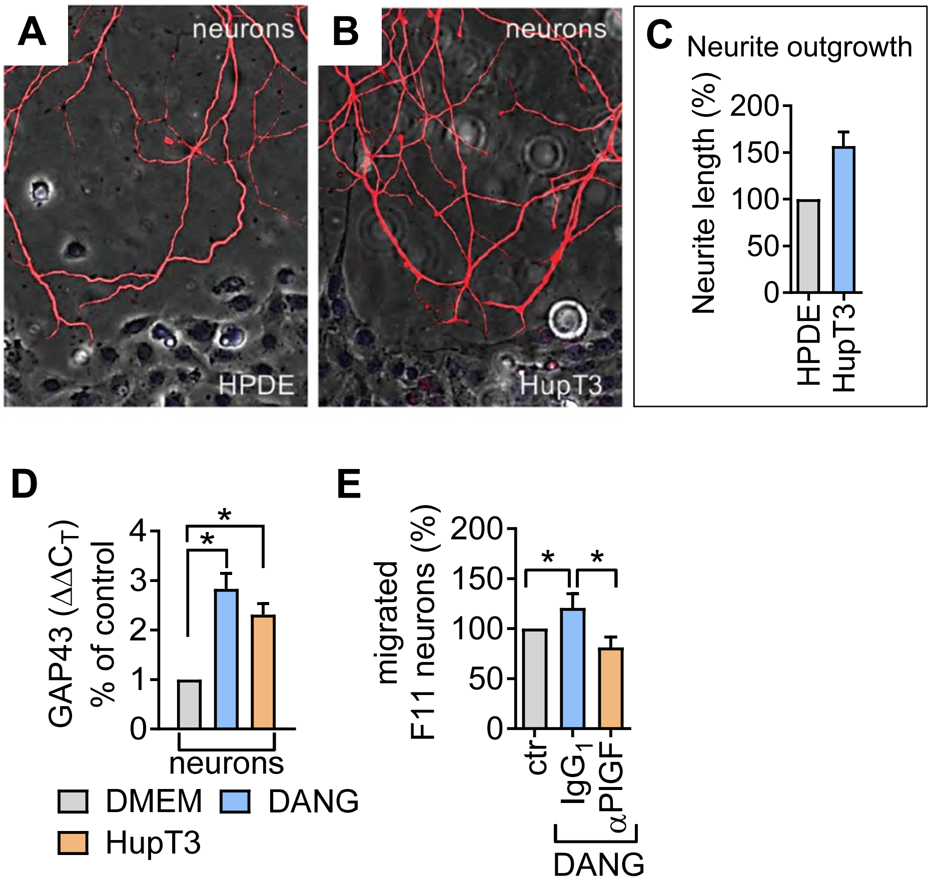

Suppl. Figure 6: *PDAC stimulates neural plasticity.*

A-C, Non-transformed HPDE cells (**A**) and HupT3 PDAC cells (**B**) were cultured with primary neurons in separate patches divided by a 500µm gap using IBIDI® inserts, and nascent neurites were visualized by immunofluorescent staining for β3-tubulin following 48 h. Neurite outgrowth was quantified by determining the neurite length and shown as mean±SEM (**C,** n=3; $p<0.05$). **D,** Conditioned media from PDAC cell lines DANG and HupT3 induce expression of the growth-associated-protein (GAP)-43 in primary neurons. Shown are mean±SEM GAP43 mRNA transcript expression in primary neurons. **E,** Blocking PlGF in conditioned media from DANG cell cultures abrogated the directed migration of F11 neurons towards chemoattractant released from DANG cell cultures. Shown are mean±SEM (n=3; $p<0.05$). *, $P<0.05$.
