# Supplementary material for: Placental growth factor promotes neural invasion and predicts disease prognosis in resectable pancreatic cancer
Source: J Exp Clin Cancer Res. 2024 May 30;43:153. doi: 10.1186/s13046-024-03066-z (PMC11138065; doi:10.1186/s13046-024-03066-z)
Supplement: Supplementary file 8 — Suppl. Material and Methods. [file 13046_2024_3066_MOESM8_ESM.docx]

**SUPPLEMENTAL MATERIAL AND METHODS**

***ELISA***

Protein lysates from xenografted tumor specimens were obtained by mechanical homogenization in cell lysis buffer (MCL-1; Sigma, Missouri, USA) as described ([25](#_ENREF_25)). Concentration of human and/or murine PlGF in human serum samples, cell culture supernatants and protein lysates were quantified using species-specific Quantikine® ELISA kits according to the manufacturer. Concentrations were expressed as pg/mg protein.

***Quantitative RT-PCR***

RNA from forty 20µm tissue slices or cultured cells was extracted and purified using the RNeasy-mini kit (Qiagen, Hilden, Germany), and RNA concentration and quality were determined on Agilent's 2100 Bioanalyzer using the RNA-6000-Nano Kit (Agilent, Santa Clara, USA) as described ([12](#_ENREF_12)). qRT-PCR was carried out in triplicate 10µl-reactions using species-specific PlGF, VEGFR1, NRP1 and GAP43 TaqMan primer/probes from Applied-Biosystems (Foster City, USA) with the One-Step RT-PCR Kit (Invitrogen) on a CFX96 thermo-cycler (Bio-Rad, Hercules, USA) and normalized based on GAPDH transcripts. RNA isolation and qRT-PCR of cultured cells were performed as described ([12](#_ENREF_12)).

***Preparation of cell extracts and immunoblotting***

5x10^6^ cells cultured in monolayers were lysed in 100µl RIPA buffer supplemented with protease inhibitors and immunoblotted as described ([12](#_ENREF_12)).

***Immunohistochemical analyses and primary antibodies***

Paraffin sections were cut at 4µm slices from dehydrated and paraffin embedded human PDAC tissues. Immunoperoxidase staining was performed using Vectastain Elite ABC-kit (Vector Laboratories, Wertheim-Bettingen, Germany) and AEC as substrate chromogen (DAKO, Hamburg, Germany). Primary antibodies included anti-Nrp1 (1:100; sc-5307, Santa Cruz Biotechnology, California, USA) and anti-β3-tubulin (clone Tuj1; #801201, BioLegend, Amsterdam, The Netherlands) and were omitted in negative controls. Secondary antibodies were from Dianova (Hamburg, Germany).
